# SLC1A5 enhances malignant phenotypes through modulating ferroptosis status and immune microenvironment in glioma

**DOI:** 10.1038/s41419-022-05526-w

**Published:** 2022-12-24

**Authors:** Liying Han, Jinpeng Zhou, Leiyang Li, Xun Wu, Yingwu Shi, Wenxing Cui, Shenghao Zhang, Qing Hu, Jin Wang, Hao Bai, Haixiao Liu, Chengxuan Guo, Haiyan Cao, Min Chao, Yaqin Hu, Yueyang Mou, Yang Jiao, Dayun Feng, Liang Wang, Yan Qu

**Affiliations:** Department of Neurosurgery, Tangdu Hospital, Fourth Military Medical University, Xi’an, China

**Keywords:** CNS cancer, Prognostic markers, Cell death

## Abstract

Glioma is the most common type of primary malignant tumor in the central nervous system with limited treatment satisfaction. Finding new therapeutic targets has remained a major challenge. Ferroptosis is a novel and distinct type of programmed cell death, playing a regulatory role in the progression of tumors. However, the role of ferroptosis or ferroptosis-related genes (FRGs) in glioma progression has not been extensively studied. In our study, a novel ferroptosis-related prognostic model, including 7 genes, was established, in which patients classified into the high-risk group had more immuno-suppressive status and worse prognosis. Among these 7 genes, we screened solute carrier family 1 member 5 (SLC1A5), an FRG, as a possible new target for glioma treatment. Our results showed that the expression of SLC1A5 was significantly upregulated in glioblastoma tissues compared with the low-grade gliomas. In addition, SLC1A5 knockdown could significantly inhibit glioma cell proliferation and invasion, and reduce the sensitivity of ferroptosis via the GPX4-dependent pathway. Furthermore, SLC1A5 was found to be related to immune response and SLC1A5 knockdown decreased the infiltration and M2 polarization of tumor-associated macrophages. Pharmacological inhibition of SLC1A5 by V9302 was confirmed to promote the efficacy of anti-PD-1 therapy. Overall, we developed a novel prognostic model for glioma based on the seven-FRGs signature, which could apply to glioma prognostic and immune status prediction. Besides, SLC1A5 in the model could regulate the proliferation, invasion, ferroptosis and immune state in glioma, and be applied as a prognostic biomarker and potential therapeutic target for glioma.

## Introduction

Glioma is the most common type of primary malignant tumor in the central nervous system with poor prognosis, especially glioblastoma (WHO IV glioma, GBM), which has a median survival time of fewer than 2 years [1]. Molecular markers, such as IDH mutation and MGMT methylation, are utilized for molecular pathological diagnosis, treatment choice, and prognostic assessment of glioma patients [2]. These molecular markers are critical in mediating glioma cell fate and have been used as therapeutic targets in numerous clinical trials, but few of them succeeded eventually [3]. Therefore, revealing the mechanism of glioma progression and identifying novel molecular targets for glioma therapy are urgently needed.

Ferroptosis has been found as a form of cell death and an important potentiator for immunotherapy [4] and chemotherapy [5]. In previous studies, ferroptosis has been verified to make impacts in several cancers, such as breast cancer [6], renal cell carcinoma [7], lung cancer [8], and pancreatic cancer [9]. Consequently, ferroptosis induction is an emerging approach to cancer therapy [10]. However, ferroptosis could trigger inflammation-associated immune responses, thus affecting tumor growth [11]. Recent research has proven that ferroptosis is one of the major types of programmed cell death in gliomas and affects the immune microenvironment in glioma patients [12]. Hence, the relationship between ferroptosis-related genes (FRGs) and the progression of glioma needs further exploration.

Solute carrier family 1 member 5 (SLC1A5) is a glutamine transporter on the cell membrane [13], acting as a ferroptosis-inducing gene in previous studies [14]. The metabolite of glutamine, α-KG, enters the TCA cycle, promotes the oxidative phosphorylation pathway, and increases the level of cellular oxidative stress [15]. SLC1A5 is highly expressed in many cancers [16], and the pharmacological blockade of SLC1A5 by V9302 for cancer treatment is now under preclinical trials [17]. Targeting SLC1A5 has been considered as a potential strategy to strengthen anti-tumor immunity [18]. However, the effect of SLC1A5 and the relationship between SLC1A5 and immune state in glioma remains unclear.

The goal of our study is to establish a ferroptosis-related prognostic model that could apply to glioma prognostic prediction and verify the molecular mechanism of the FRG, SLC1A5, in glioma. We summarized the data of glioma patients from public databases and identified a model of seven FRGs associated with glioma prognosis. In addition, functional enrichment and immuno-infiltration analyses were performed to explore potential mechanisms. After screening and validation, we revealed that SLC1A5 mediated the malignant phenotypes by affecting the ferroptosis state, the infiltration and polarization of tumor-associated macrophages (TAMs) in glioma.

## Results

### Establishment of an FRGs-related prognostic model in public databases

**Fig. 1:**
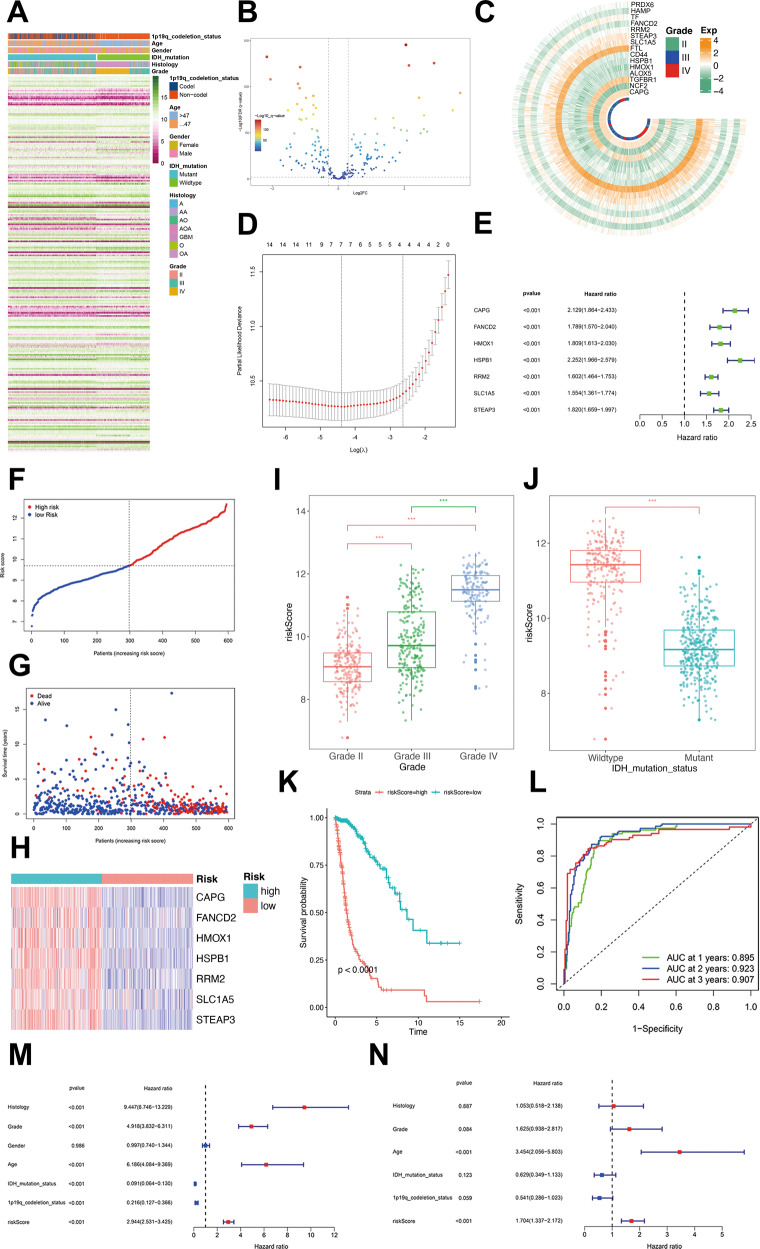
Establishment of an FRGs-related prognostic model in the TCGA cohort. **A** The expression of the total 242 FRGs identified from FerrDb in the TCGA cohort. **B** The differentially expressed FRGs between GBM and low-grade glioma patients. **C** The expression of 15 independent prognosis ferroptosis-related DEGs in TCGA cohort. **D** The prognostic model was constructed by 15 FRGs through LASSO regression analysis. **E** Univariate-Cox regression analysis of each gene in the model. **F** Patients were divided into high-risk and low-risk groups. **G** The relationship between survival state and risk score. **H** The expression of seven FRGs of the model in the high-risk group and low-risk groups. **I** The relationship between the risk score and WHO grade. **J** The relationship between the risk score and IDH mutation status. **K** Kaplan–Meier survival analysis for the survival time of high-risk patients and low-risk patients. **L** The ROC curves and AUC of the risk score in TCGA datasbase. Univariate-(**M**) and multivariate-(**N**) Cox regression analysis of the risk score.

Total 242 FRGs were identified from FerrDb following the exclusion criteria (Fig. 1A). First, the differentially expressed genes (DEGs) filter was conducted to select the FRGs that were significantly differentially expressed between GBM and low-grade glioma patients in TCGA cohort (|LogFoldchange| > 0.3, *P* < 0.01). Thus, 125 FRGs were differentially expressed (Fig. 1B). Independent prognosis analysis was then conducted to screen for the ferroptosis-related DEGs of other factors affecting patient’s survival, such as WHO grade, age, histological subtypes, and IDH types. In these 125 ferroptosis-related DEGs, we identified only 15 DEGs with independent prognosis (*P* < 0.001; Fig. 1C). A seven-genes signature model was established by LASSO regression, which included capping actin protein, gelsolin like (CAPG), Fanconi anemia complement group D2 (FANCD2), heme oxygenase-1(HMOX1), heat shock protein beta-1(HSPB1), ribonucleotide reductase regulatory subunit M2(RRM2), SLC1A5, and six-transmembrane epithelial antigen of prostate 3 (STEAP3) (Fig. 1D). Coefficients of each gene in the model were shown in Supplementary Table 1. According to the univariate-Cox regression, each gene in the model was a risk factor for the prognosis (Fig. 1E). Patients were divided into high-risk and low-risk groups according to the median risk score (Fig. 1F). Patients with poor prognosis mainly showed higher risk scores (Fig. 1G) and the expression of seven FRGs was higher in the high-risk group than that in the low-risk group (Fig. 1H). The risk score was also increased with the WHO grade of glioma (Fig. 1I) and higher in IDH wild-type group than that in IDH mutant group (Fig. 1J). Patients in the high-risk group had significantly shorter survival time than those in the low-risk group (Fig. 1K). The AUC of the risk score in TCGA cohort suggested that the model had favorable predictive value in both short and long-term follow-ups of glioma patients (Fig. 1L). The univariate and multivariate Cox regression analysis confirmed that the risk score was an independent predictor of survival in TCGA cohort (Fig. 1M, N). The reliability of the risk score was also verified in other validation cohorts: CGGA-325 (Supplementary Fig. S1A–F), CGGA-639 (Supplementary Fig. S1G–L), and Rembrandt (Supplementary Fig. S1J–Q).

In previous studies, ferroptosis could affect immunosuppression in tumor microenvironment [11]. Therefore, we validated the relationship between the model and the immunosuppressive microenvironment. The association between risk score and immune-related biological processes was clearly displayed by GO analysis (Supplementary Fig. S2A, B). The CIBERSORT results showed that patients in the high-risk group exhibited significant enrichment of immune-infiltration cells, especially M2-like macrophages (Supplementary Fig. S2C). The ssGSEA results demonstrated that the immune-cell scores and related pathways were significantly higher in the high-risk group than in the low-risk group in TCGA cohort (Supplementary Fig. S2D, E). Furthermore, we found that patients in the high-risk group had higher immune scores, stromal scores, and lower tumor purity (Supplementary Fig. S2F–H). The above results suggested that patients in the high-risk group might have a more immunosuppressive tumor microenvironment and might not benefit from immune checkpoint blockade therapy.

### The expression of SLC1A5 was increased with glioma grades and correlated with poor prognosis

**Fig. 2:**
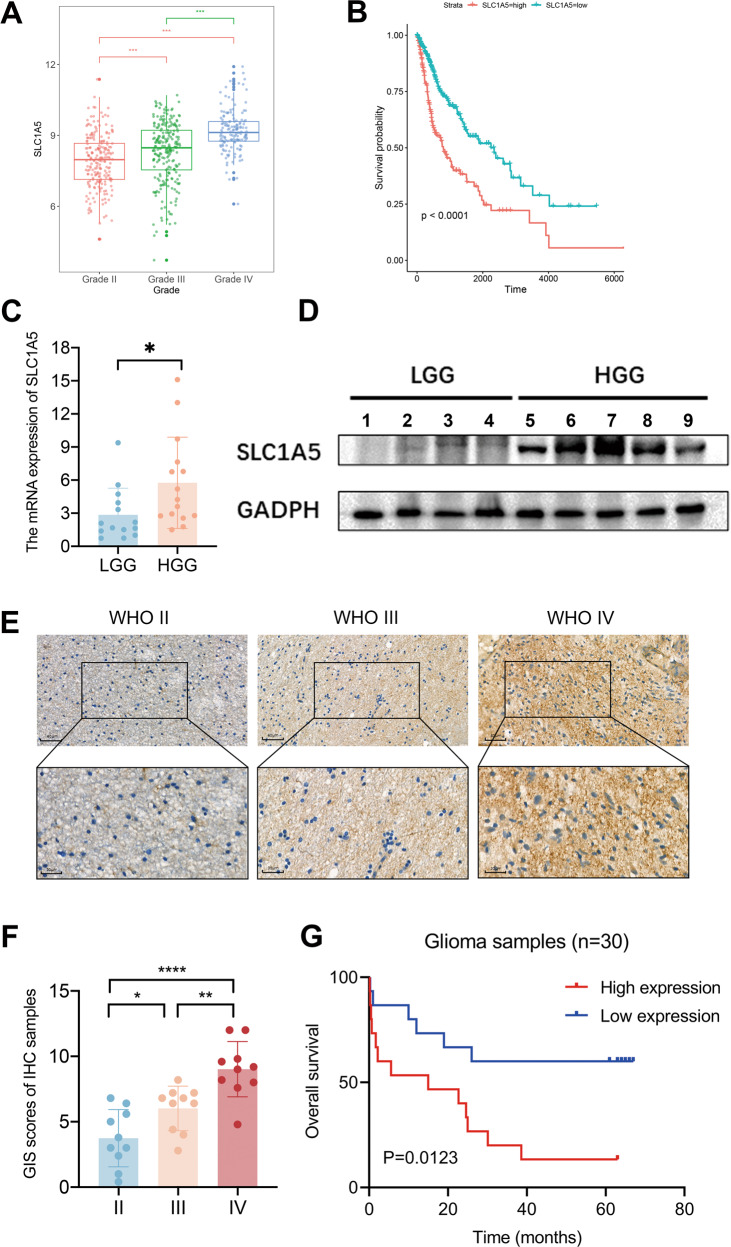
High expression of SLC1A5 was assessed in glioblastoma and associated with poor survival outcomes of glioma. **A** The relationship between the expression of SLC1A5 and WHO grade in TCGA cohort. **B** Kaplan–Meier survival curves of the patient’s survival time in SLC1A5 high- or low-expression groups in TCGA cohort. The qPCR (**C**) and WB (**D**) results showing the expression of SLC1A5 in Tangdu glioma cohort. **E**, **F** IHC staining and scores of the expression of SLC1A5 in Tangdu glioma cohort. **G** Kaplan–Meier survival curves of the patient’s survival time in SLC1A5 high- or low-expression groups from Tangdu cohort.

Bioinformatics analysis of public databases revealed that all seven genes in the model correlated with the WHO grade and prognosis of glioma patients (Fig. 2A, B, Supplementary Figs. S3–5). However, previous studies of CAPG, FANCD2, HMOX1, HSPB1, RRM2, and STEAP3 did not develop any new clinical therapy in glioma [19–25]. Therefore, we hypothesized that SLC1A5 might function in glioma progression and clinical treatment.

To confirm the results of bioinformatic analysis, further WB, qPCR, and IHC were conducted to examine the expression of SLC1A5 in glioma tissues from Tangdu hospital. The qPCR and WB results demonstrated the expression of SLC1A5 was increased in high-grade gliomas (Fig. 2C, D). Consistently, the IHC staining indicated that SLC1A5 was highly expressed in glioblastoma (Fig. 2E, F). Kaplan–Meier survival analysis showed the expression of SLC1A5 was negatively associated with survival outcomes in Tangdu cohort (Fig. 2G). These results suggested that high expression of SLC1A5 was mainly found in GBM and predicted a poor prognosis for glioma patients.

### SLC1A5 regulated the proliferation and invasion of glioma cells in vitro

To investigate the function of SLC1A5 in vitro, we first examined the protein and mRNA expression of SLC1A5 in human normal astrocyte cell line HA1800 and different glioma cell lines and noticed that the expression of SLC1A5 was higher in the glioma cells (Supplementary Fig. S6). The expression of SLC38A1, another glutamine transporter on the cell membrane, was less different in these cell lines (Supplementary Fig. S6A), suggesting SLC1A5 was the main glutamine transporter in glioma cells. To investigate the function of SLC1A5 in glioma cells, we selected the T98G cell line for the following cytological experiments due to its significant expression of SLC1A5. We performed the transfection of lentivirus with SLC1A5 overexpression and knockdown vector in the T98G cells. The transfection efficacy was validated by WB and qPCR (Supplementary Fig. S7).

**Fig. 3:**
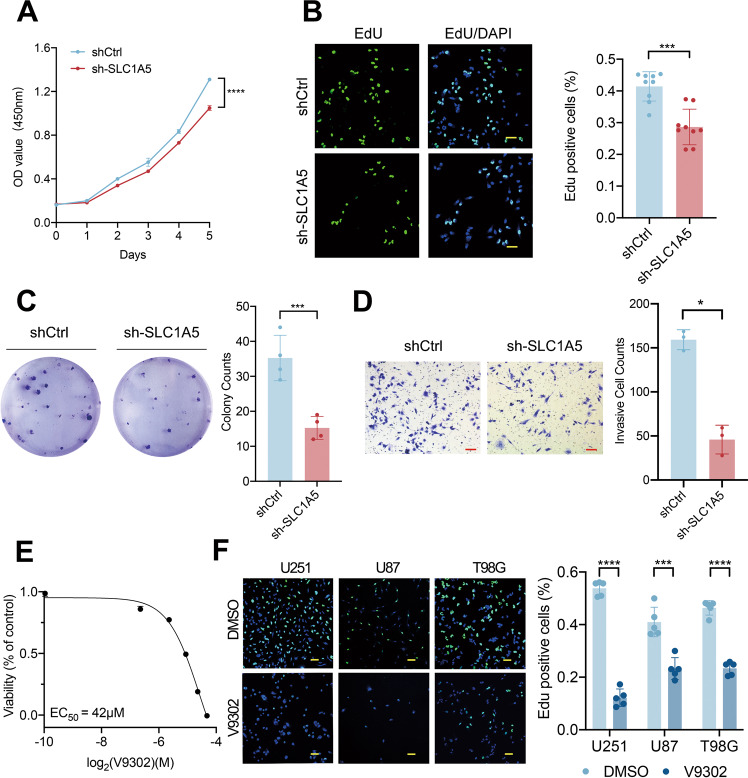
SLC1A5 regulated the proliferation and invasion of glioma cells in vitro. **A** The CCK-8 assay results of cell viability after SLC1A5 knockdown. **B** The EdU assay results of proliferative phase cells after SLC1A5 knockdown (Scale Bar = 20 μm). **C** The colony formation results of T98G cells after SLC1A5 knockdown. **D** The transwell assay results of invasive cells after SLC1A5 knockdown (Scale Bar = 20 μm). **E** EC50 of V9302 in T98G cells. **F** The EdU assay results of proliferative phase cells after V9302 treatment in U251, U87 and T98G cells (Scale Bar = 20 μm).

To validate the proliferative and invasive potential of SLC1A5, the CCK-8, EdU, and colony formation and transwell invasion assays were conducted in transfected T98G cells. The CCK-8 assay showed that the cell viability of sh-SLC1A5 group was lower than that of shCtrl group (Fig. 3A). As tested by the EdU and colony formation assays, SLC1A5 knockdown significantly decreased the proliferation and growth of the T98G cells (Fig. 3B, C). In the transwell assay, the invasive cells in sh-SLC1A5 group were decreased, compared with those in shCtrl group (Fig. 3D). On the contrary, SLC1A5 overexpression in T98G cells significantly promoted the cell viability, proliferation and invasion of glioma cells (Supplementary Fig. S8). V9302 was used to treat the glioma cells and the concentration for 50% of maximal effect (EC50) of V9302 in T98G cell was 42μmol (Fig. 3E). V9302 could suppress the proliferation of the glioma cells (U251, U87, and T98G) by EdU assay (Fig. 3F). These results suggested that SLC1A5 could significantly facilitate the malignant phenotype of glioma cells.

### SLC1A5 modulates the tumorigenesis of glioma in vivo

**Fig. 4:**
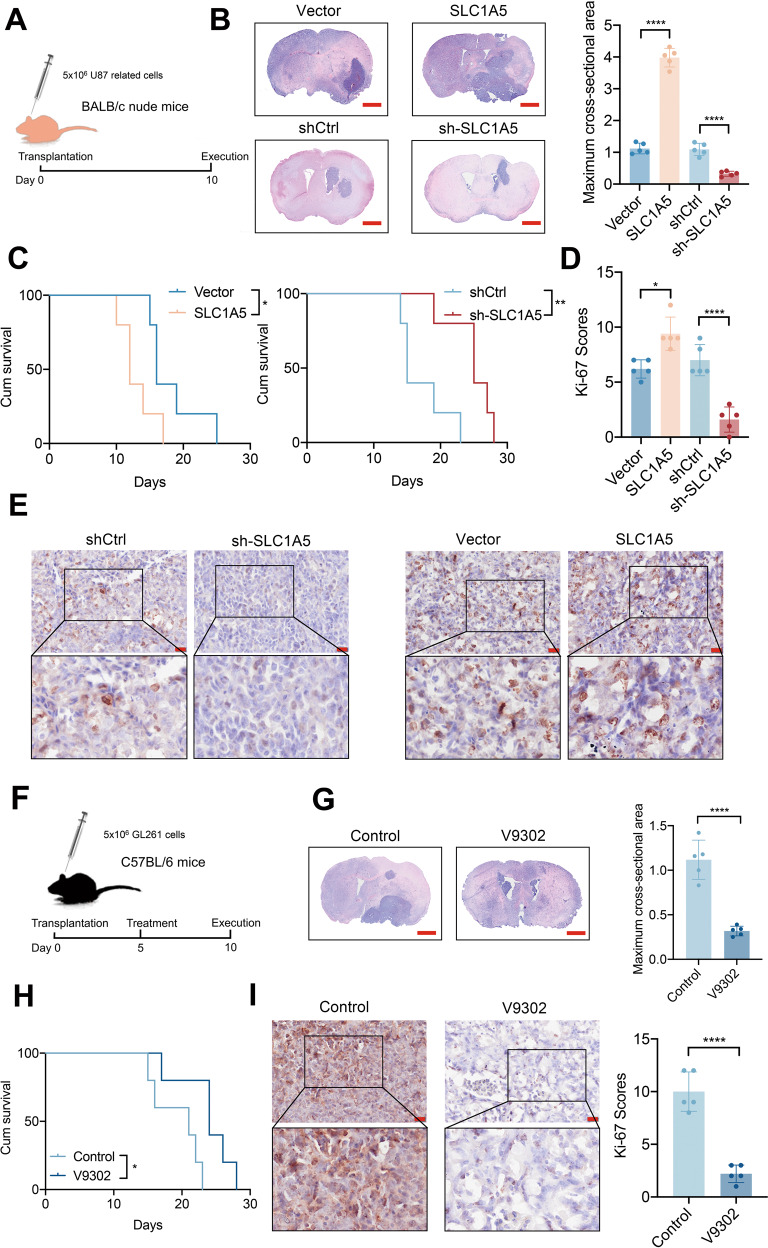
SLC1A5 modulated the glioma growth in vivo. **A** The schematic illustration of U87 related cells transplantation in BALB/c nude mice. **B** The HE staining and the tumor size analysis for mice in SLC1A5 overexpression or knockdown groups (Scale Bar = 1 mm). **C** Kaplan–Meier survival curves of the mice survival time in SLC1A5 overexpression or knockdown groups. **D** The Ki-67 scores of glioma sections in SLC1A5 overexpression or knockdown groups. **E** The Ki-67 IHC images of glioma sections in SLC1A5 overexpression or knockdown groups (Scale Bar = 20 μm). **F** The schematic illustration of V9302 treatment in GL261-bearing C57BL/6 mice. **G** The HE staining and the tumor size analysis for mice in the control and V9302 groups (Scale Bar = 1 mm). **H** Kaplan–Meier survival curves of the survival time of mice in the control and V9302 groups. **I** The Ki-67 IHC images and scores of glioma tissues in the control and V9302 groups (Scale Bar = 20 μm).

Considering that SLC1A5 affected the proliferation and invasion of glioma cells in vitro and the susceptibility of U87 cells to tumor formation in the brain of nude mice, we transplanted the transfected U87 cells into the brains of BALB/c nude mice respectively (Fig. 4A). HE staining demonstrated that the tumor size in SLC1A5 group was significantly larger than that in vector group, and glioma in sh-SLC1A5 group, by contrast, developed more slowly than that in shCtrl group (Fig. 4B). Mice in SLC1A5 group had a shorter survival time, while mice in sh-SLC1A5 group had a better prognosis (Fig. 4C). IHC results showed higher Ki-67 scores in SLC1A5 group and lower Ki-67 scores in sh-SLC1A5 group (Fig. 4D, E). We also verified the effect of V9302 on glioma in vivo (Fig. 4F). HE staining showed that the tumor size of the control group was significantly larger than that of the V9302 group (Fig. 4G). Mice in the V9302 group had a better prognosis than that in the control group (Fig. 4H). The IHC results indicated that Ki67 scores of tumor tissues in the V9302 treatment group were lower (Fig. 4I). The above results suggested that SLC1A5 had a growth-promoting effect on glioma in vivo, which were consistent with in vitro experiment results.

### SLC1A5 inhibits the cellular oxidative stress damage and the ferroptosis state via the GPX4-related pathway

**Fig. 5:**
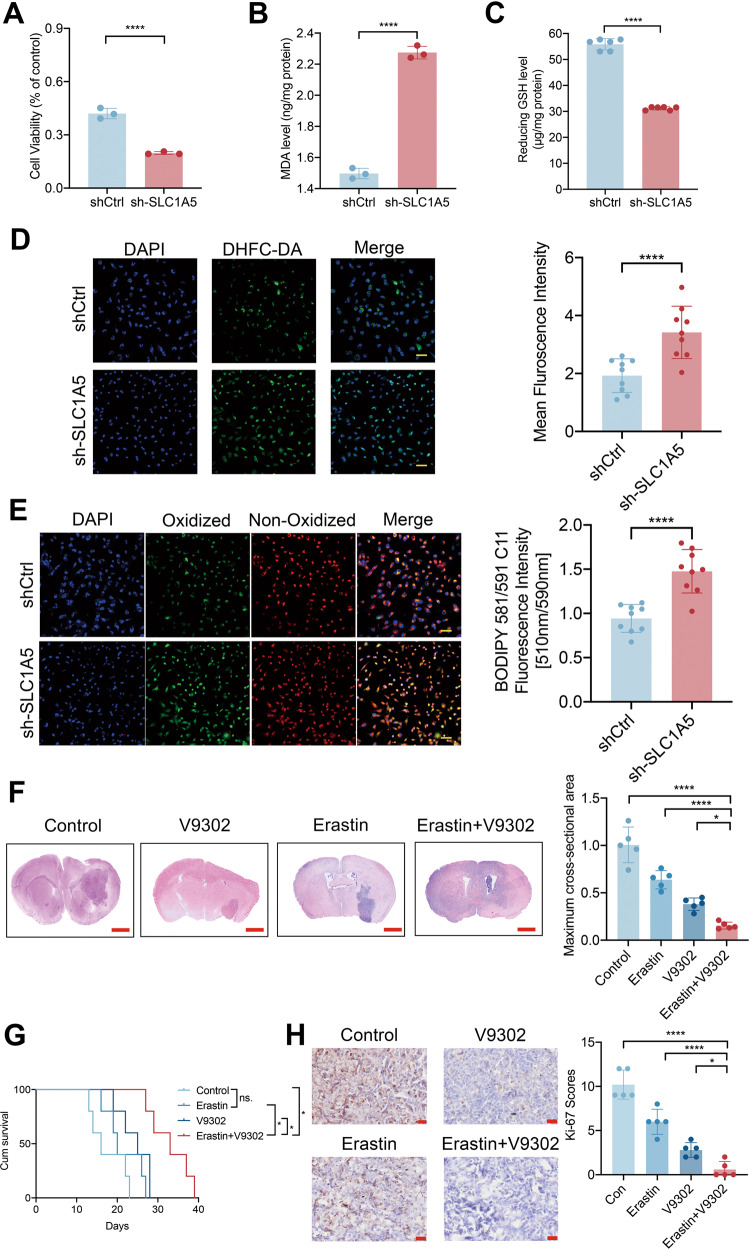
SLC1A5 resisted cellular oxidative stress damage and ferroptosis state in glioma cells. **A** The CCK-8 assay results of cell viability after SLC1A5 knockdown with Erastin treatment. **B** The MDA level after SLC1A5 knockdown with Erastin treatment. **C** The level of reduced GSH after SLC1A5 knockdown with Erastin treatment. **D** The fluorescence intensity of DHFC-DA (green) in SLC1A5 knockdown T98G cells with Erastin treatment (Scale Bar = 20 μm). **E** The level of oxidized lipid peroxides(green) in SLC1A5 knockdown T98G cells with Erastin treatment (Scale Bar = 20 μm). **F** The HE staining and the tumor size analysis in Erastin and V9302 separate and combined treatment groups (Scale Bar = 1 mm). **G** Kaplan–Meier survival curves of survival time in different treatment groups. **H** The Ki-67 IHC images and scores of glioma sections in different treatment groups (Scale Bar = 20 μm).

Ferroptosis is an iron-dependent, oxidative form of regulated cell death, characterized by the accumulation of lipid peroxides, elevated ROS level, and reduced intracellular glutathione (GSH) level [26]. In our study, transfected T98G cells were treated with 20 µM Erastin, a classic ferroptosis inducer. After treatment for 12 h, we found that the cell viability in sh-SLC1A5 group was significantly lower than that in shCtrl group (Fig. 5A), while the cell viability in SLC1A5 group was significantly higher by CCK-8 assay (Supplementary Fig. S9A). In addition, after Erastin treatment, we found that cells in sh-SLC1A5 group had a higher level of MDA and a lower level of reduced GSH compared with those in shCtrl group, while cells in SLC1A5 group had a lower level of MDA and higher level of reduced GSH (Supplementary Fig. S7B, C). Confocal images of ROS and LPO in the cells showed the sh-SLC1A5 T98G cells exhibited a stronger fluorescence intensity of ROS and LPO (Fig. 5D, E), while the cells in SLC1A5 group had a lower fluorescence intensity (Supplementary Fig. S9D, E). Pharmacological inhibition of SLC1A5 also increased the MDA level (Supplementary Fig. S10A), decreased the reduced GSH (Supplementary Fig. S10B), and strengthened the fluorescence intensity of ROS and LPO probes (Supplementary Fig. S10C–F).

Five days after GL261 transplantation, V9302 and Erastin were injected separately or in combination intraperitoneally for 5 days. HE staining showed that the tumor sizes were the smallest in the combined treatment group (Fig. 5F). Mice in the V9302 and Erastin co-treatment group had the longest survival time and best prognosis than those in the other groups (Fig. 5G). The IHC results showed the lowest Ki-67 scores in the co-treatment group. (Fig. 5H). These results indicated that V9302 promoted the anti-tumor efficacy of Erastin in vivo. Based on the above results, we inferred that SLC1A5 was able to resist oxidative stress damage and ferroptosis state. The combination of ferroptosis inducer with the inhibition of SLC1A5 could further inhibit glioma growth.

**Fig. 6:**
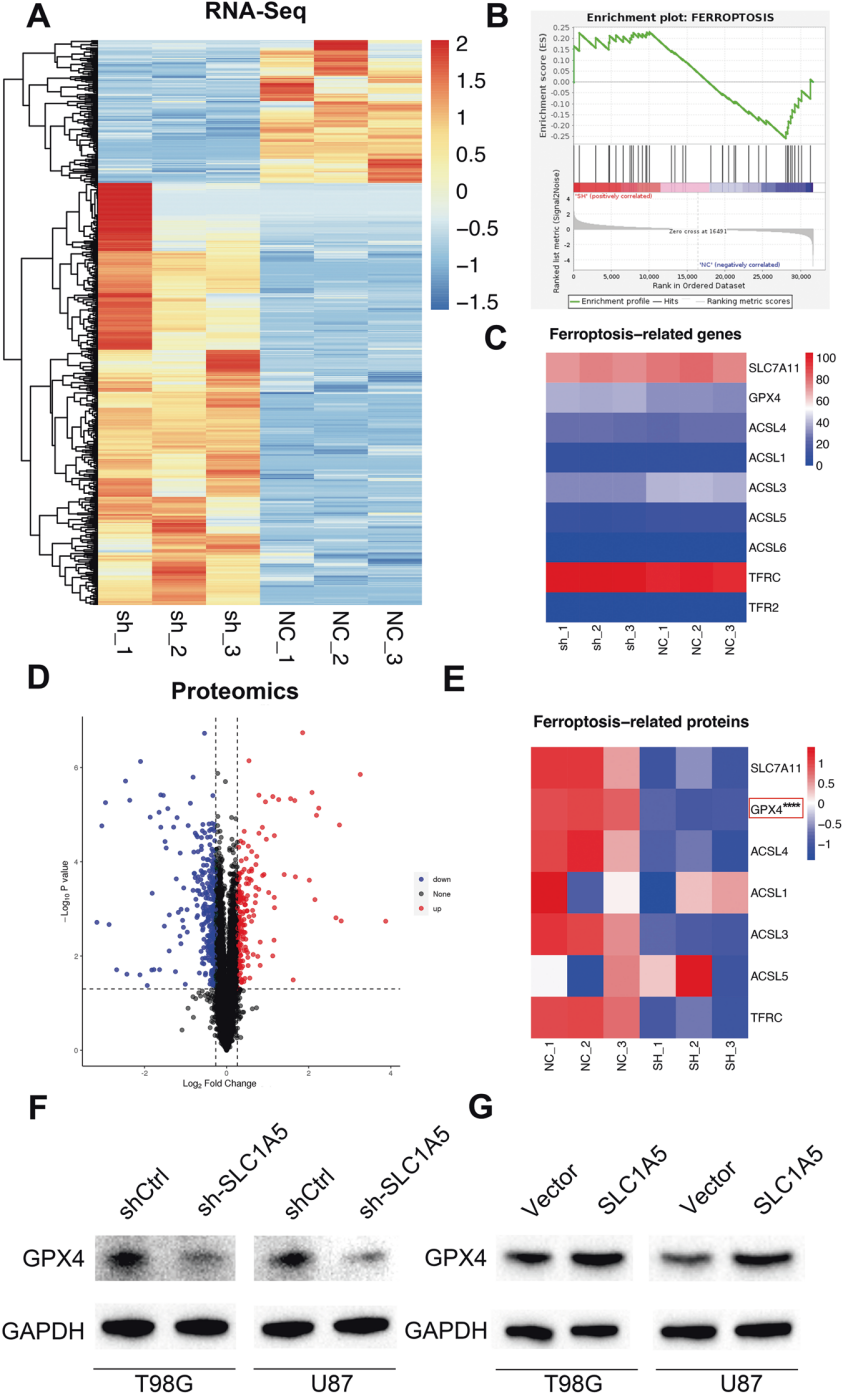
Integrated proteotranscriptomics analysis revealed that SLC1A5 regulated ferroptosis state by affecting the expression of GPX4. **A** The differentially expressed genes between shCtrl and sh-SLC1A5 groups through RNA-sequence. **B** GSEA analysis showed the correlation between the differentially expressed genes and ferroptosis pathway. **C** The mRNA expression of ferroptosis-related genes in the shCtrl and sh-SLC1A5 group. **D** The differentially expressed proteins between shCtrl and sh-SLC1A5 group through TMT-proteomics analysis. **E** The expression of ferroptosis-related proteins in the shCtrl and sh-SLC1A5 group. The Western Blot indicating the expression of GPX4 in T98G and U87 cell lines after SLC1A5 knockdown (**F**) or overexpression (**G**) by Western Blot.

We performed transcriptome sequencing after SLC1A5 knockdown in the T98G cells (Fig. 6A), and found that the DEGs between shCtrl and sh-SLC1A5 groups were mainly enriched in the ferroptosis pathway through GSEA analysis (Fig. 6B). However, the mRNA expression of ferroptosis-related genes had no significant difference between two groups (Fig. 6C). Next, proteomic sequencing between shCtrl and sh-SLC1A5 groups was conducted and the differentially expressed proteins were analyzed (Fig. 6D). Among the ferroptosis-related proteins, we found that only GPX4 (a negative regulator of ferroptosis) expression was significantly downregulated in sh-SLC1A5 group (Fig. 6E). Furthermore, WB results showed GPX4’s expression was decreased in sh-SLC1A5 group (Fig. 6F), while it was increased in SLC1A5 group (Fig. 6G) in T98G and U87 cells. The above results suggested that high expression of SLC1A5 in glioma cells could increase GPX4 expression, thus suppressing intracellular oxidative stress and ferroptosis level.

### SLC1A5 inhibition decreased the recruitment and M2 polarization of TAMs and elevated the efficacy of immunotherapy

**Fig. 7:**
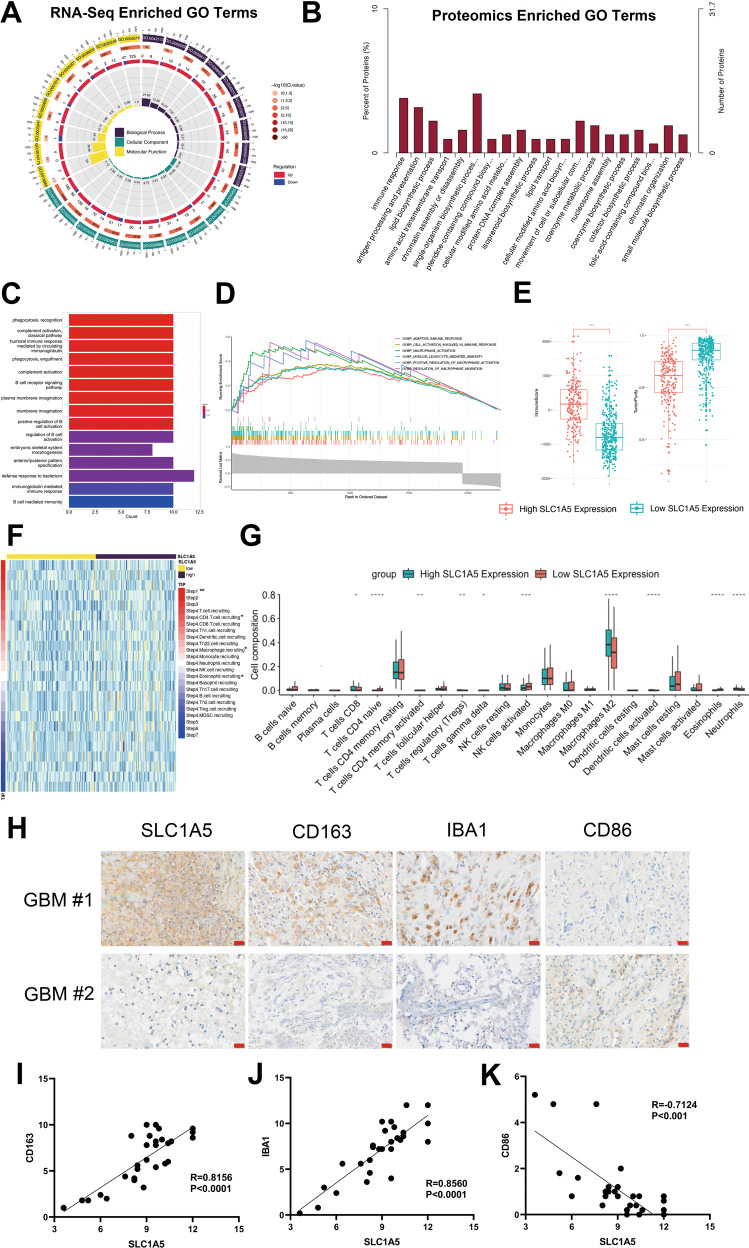
SLC1A5 was associated with the infiltration of TAMs in glioma tissues. **A** GO enrichment analysis of DEGs between shCtrl and sh-SLC1A5 groups through RNA-sequence. **B** GO enrichment analysis of differentially expressed proteins between two groups through proteomics analysis. **C** GO enrichment analysis of the SLC1A5-related genes in TCGA cohort. **D** GSEA enrichment analysis of the SLC1A5-related genes in TCGA cohort. **E** The association of immune scores, tumor purity and SLC1A5 expression in TCGA cohort. **F** The relationship between tumor immunophenotype and SLC1A5 expression in TCGA cohort. **G** CIBERSORT analysis of the infiltration of immune cells between SLC1A5 high- and low-expression group in TCGA cohort. **H** The IHC results showing the expression of CD163, IBA-1 and CD86 in GBM tissues with different expression of SLC1A5 (Scale Bar = 20 μm). **I–K** The correlation between the expression of SLC1A5 and the expression of CD163 (**F**), IBA-1 (**G**) or CD86 (**H**) in GBM tissues.

The main role of SLC1A5 is to transport extracellular glutamine into the cytoplasm. Previous studies had demonstrated that glutamine regulated the function and differentiation of immune cells [27]. GO enrichment analysis of transcriptome and proteome sequencing in shCtrl and sh-SLC1A5 cells revealed that the differentially expressed genes or proteins were focused on immune-related pathways, such as immune response and antigen processing and presentation (Fig. 7A, B). The relationship between SLC1A5 and immunity was verified in TCGA database. The GO and GSEA enrichment analysis showed that SLC1A5 was associated with the activation and infiltration of immune cells, especially macrophages (Fig. 7C, D). Glioma patients in the SLC1A5 high-expression group had higher immune scores and lower tumor purity (Fig. 7E). The analysis was performed to reflect the status of anti-tumor immunity and the results showed that although SLC1A5 mediated antigen activation (Step 1) and motivated a variety of immune cells including T cells and macrophages (Step 4), it had no role in T cell recognition and tumor killing (Step 6 and 7) (Fig. 7F). Therefore, it indicated that SLC1A5 suppressed anti-tumor immunity despite increased immune cell mobilization. CIBERSORT result also showed that patients in the SLC1A5 high-expression group had more significant infiltration of M2-polarized macrophages than those in the SLC1A5 low-expression group (Fig. 7G). Given the above results, we speculated that SLC1A5 could regulate the infiltration and state of TAMs in glioma microenvironment.

**Fig. 8:**
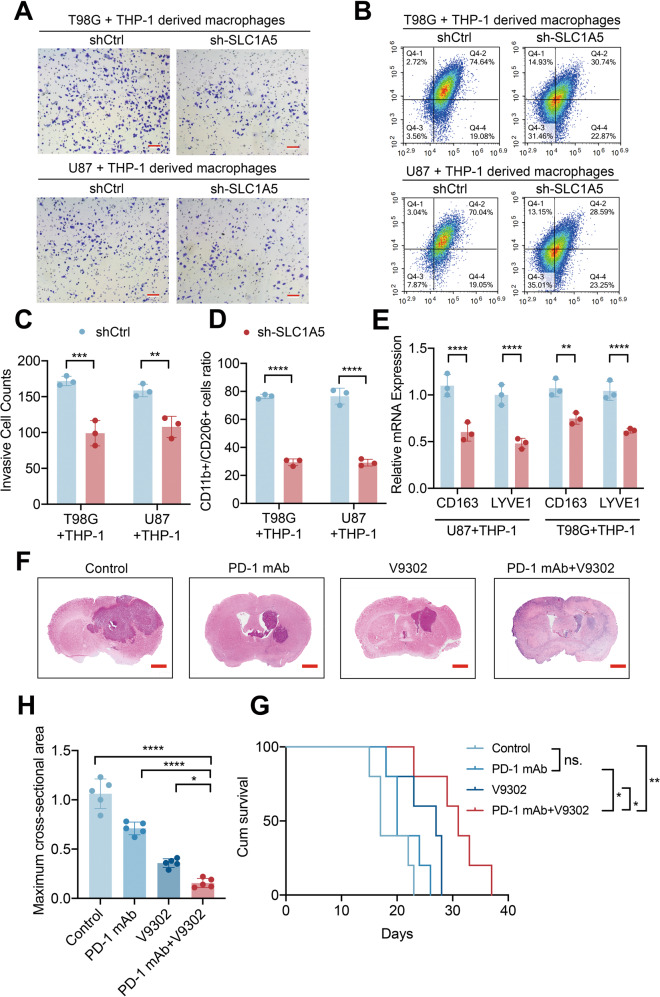
SLC1A5 modulated THP-1 derived macrophages infiltration and its inhibitor synergized with immune checkpoint inhibitor to suppress tumor growth. **A**, **C** The migration of THP-1 derived macrophages co-cultured with different groups of GBM cells (Scale Bar = 20 μm). **B**, **D** Flow cytometry results representing the percent of CD11b^+^ CD206^+^ macrophages after co-cultured with different groups of GBM cells. **E** Relative mRNA expression of CD163 and LYVE1 (M2 macrophage markers) in THP-1 derived macrophages co-cultured with different groups of GBM cells. **F**–**H** The HE staining and tumor size analysis of mice in PD-1mAb and V9302 separate or combined treatment group (Scale Bar = 1 mm). **G** The survival time of GL261-bearing C57BL/6 mice under different treatments.

TAMs are the most enriched immune cells in GBM and trigger proinflammatory (M1) or immunosuppressive (M2) responses. We performed IHC experiment on GBM tissues and identified that the expression of SLC1A5 was positively correlated with the expression of CD163 (M2-like macrophage marker) and IBA-1 (total macrophage marker), whereas negatively associated with CD86 expression (M1-like macrophage marker) (Fig. 7H–K). To further detect the effects of SLC1A5 knockdown in glioma cells on macrophages, we co-cultured THP-1 derived macrophages (PMA, 5 nM, 48 h) with shCtrl and sh-SLC1A5 glioma cells. The transwell assay showed that the migrated cells of THP-1 derived macrophages co-cultured with sh-SLC1A5 glioma cells were significantly reduced (Fig. 8A, C). Flow cytometry showed that when the THP-1 derived macrophages were co-cultured with the sh-SLC1A5 glioma cells, the ratio of M2-like macrophages (CD11b+CD206+) were decreased (Fig. 8B, D). M2 markers (CD163 and LYVE1) expression were decreased in THP-1 derived macrophages co-cultured with sh-SLC1A5 cells as determined by qPCR (Fig. 8E). Above results suggested that SLC1A5 contributed to the chemotaxis and M2 polarization of TAMs.

Immunotherapy involving PD-1 blockade has been confirmed as a novel advance in cancer treatment [28], but the benefits in glioma were limited by immune-suppressive microenvironment [29]. Thus, we further investigated whether SLC1A5 inhibitor could sensitize glioma to immunotherapy in vivo. We performed further experiments in GL261-bearing C57BL/6 mice. After GL261 cells were transplanted for 5 days, PD-1 mAb and V9302 were injected intraperitoneally separately or in combination for 6 days. HE staining showed that tumor sizes of mice in the co-treatment group were significantly smaller (Fig. 8F–H). Mice in the PD-1 mAb and V9302 co-treatment group had the longest survival time and best prognosis (Fig. 8G). These findings illustrated the potential immunotherapeutic benefits of glutamine transporter SLC1A5 inhibition.

## Discussion

Glioma is a molecularly heterogeneous malignancy with limited therapeutic strategies, especially for GBM [30]. Recently, molecular features have been extensively explored [31]. Therefore, identifying the key biomarkers and targets that affect the prognosis is crucial to improve the clinical prognosis of glioma patients. There is growing evidence that ferroptosis is critical in tumorigenesis and cancer therapy [32]. In previous studies, the ferroptosis-related gene signature had shown both biological and clinical significance, predicting glioma cell death and the patient’s prognosis [33]. It was verified that the signature was associated with immune checkpoint molecules in glioma and could evaluate the immunotherapy in glioma patients [34, 35]. Our study affirmed the significance of the FRG signature in glioma progression and immunosuppression microenvironment. Besides, the number of genes in our model was smaller compared with previous studies without affecting the prognostic accuracy, which increased practical feasibility.

There were six genes in the model that were previously investigated in glioma. In previous studies, the expression of CAPG in glioma was high, and increased along with the severity of the disease [19]. The expression of CAPG was obviously related to the prognosis and immune infiltration [20]. FANCD2 was correlated with glioma grade, and pharmacological inhibition of the pathway sensitized glioma cells to chemotherapeutic agents [21]. HSPB1 enhanced SIRT2-mediated G6PD activation and promoted glioma cell proliferation [22]. RRM2 contributed to the proliferation and migration of glioma cells through the ERK1/2 and AKT pathways [23]. HMOX1 and STEAP3 were associated with growth and invasion of GBM cells [24, 25]. However, above studies did not develop and apply clinical therapies targeting these genes. It might be due to the fact that these genes did not have a significant effect on the progression of gliomas. Accordingly, we proposed that SLC1A5 could play a role in the progression and clinical therapy of glioma.

SLC1A5 is a cell surface solute-carrying transporter mediating the uptake of glutamine [36]. High SLC1A5 expression has been correlated with poor survival in various types of cancer, including hepatocellular carcinoma [37], lung cancer [13], breast cancer [38], colon cancer [39] and head and neck squamous cancer [40]. Targeting glutamine metabolism pharmacologically had been proven beneficial in other cancers, such as hepatocellular carcinoma and triple-negative breast cancer [41, 42]. However, a comprehensive characterization of SLC1A5 had not yet been explored in glioma. Due to the special feature of the central nervous system and glutamine being essential for neurotransmitter synthesis [43], pharmacological glutamine inhibition should strictly control the dose and pay close attention to the side effects. In our study, we demonstrated that SLC1A5 was an oncogene in glioma, and overexpressed in glioblastoma. Inhibition of SLC1A5 by genetic interference or chemical drugs could suppress the growth of glioma in vitro and in vivo.

In previous studies, glutamate produced by glutamine catabolism is broken down through the TCA cycle, leading to the production of high level of ROS and oxidizable lipids, which promote iron toxicity [14]. However, in our study, we found that SLC1A5 was the suppressor of ferroptosis in glioma and overexpression of SLC1A5 could increase the expression of GPX4 [44]. The combination of ferroptosis inducer with the inhibition of SLC1A5 could further increase the level of oxidative stress in glioma. The inhibitory effect of SLC1A5 on ferroptosis might also be caused by excessive cellular uptake of glutamine, which could subsequently form reducing substances. The high amount of glutamine transported by SLC1A5 could provide excessive energy for glioma cell growth and proliferation, through TCA cycle as well as oxidative phosphorylation [15], under Warburg effect, in which tumor metabolism prefers anaerobic glycolysis [45]. However, tumor hypermetabolic conditions could produce oxidative stress damage, affecting tumor growth [46]. The high expression of SLC1A5 could reduce oxidative stress damage under tumor hypermetabolism through increasing the expression of GPX4, which further accelerates cell proliferation and malignant progression.

The level of glutamine in both cytoplasm and microenvironment could affect both cell proliferation and immune cell responses [28]. The association of SLC1A5 with immune cell infiltration and polarization had also been demonstrated and targeting SLC1A5 could be a potential strategy to strengthen anti-tumor immunity in previous studies [18]. In our study, transcriptome and proteome sequencing results and bioinformatic analysis in TCGA cohort showed that differential expression of SLC1A5 could affect immune cell infiltration and immune-related pathways in glioma cells. Biological experiments also verified that SLC1A5 could affect the infiltration and polarization of immune tumor-associated macrophages in tumor samples and glioma cells. SLC1A5 inhibitor combined with an immune checkpoint inhibitor could synergistically relieve tumor immunosuppression and inhibit tumor growth. However, the concrete mechanism of how the expression of SLC1A5 affects the immune response in glioma needs further investigation.

Altogether, a novel ferroptosis-related prognostic model in glioma was established and validated. The performance of prognostic model was proved to be satisfying. This model is capable of independently predicting the prognosis and immune state of glioma patients. Furthermore, SLC1A5, one of the FRGs in prognostic model, enhances the malignancy of gliomas, modulates the tumor ferroptosis status and immune microenvironment, and may be a potential prognostic biomarker and promising candidate target for glioma treatment.

## Materials and methods

### Data

All datasets used in this study are publicly available. RNA-seq data and clinical information of patients were acquired from the Cancer Genome Atlas (TCGA), Chinese Glioma Genome Atlas (CGGA), and the Repository of Molecular Brain Neoplasia Data (Rembrandt) databases. The list of FRGs was downloaded from the FerrDb web portal. In total, 259 FRGs were characterized with the following three functions: driver, suppressor, and marker. The exclusion criteria are: (1) repeated items in three functions; (2) non-coding RNA; (3) the gene symbol has no name or HUGO Gene Nomenclature Committee ID in the FerrDb database.

### Patient specimens

Paraffin-embedded glioma samples (acquired from 2016/7 to 2017/1) with WHO II (*n* = 10), WHO III (*n* = 10), WHO IV (*n* = 10), and frozen glioma samples (obtained from 2020/1 to 2020/6) were obtained from the Department of Neurosurgery, Tangdu Hospital (Xi’an, China). These patients were not treated with radiotherapy or any anti-tumor drugs prior to the surgery. All patients had signed a written informed consent, and the protocol was also approved by the Ethics Committee of Tangdu Hospital of the Fourth Military Medical University. The basic information of the patients is shown in Supplementary Table 2.

### Cell culture

Human glioma cell lines U87, T98G, LN229, U373, human astrocyte cell line HA1800, and human mononuclear macrophage line (THP-1) were purchased from Procell Life Science & Technology (Wuhan, China). U251 was purchased from the Shanghai Cell Bank of the Chinese Academy of Sciences (Shanghai, China). Murine glioma cell line GL261 was obtained from American Type Culture Collection (Manassas, VA, USA). All cell lines were tested without mycoplasma contamination. The human and murine glioma cells were cultured in Dulbecco’s Modified Medium (DMEM) with 10% fetal bovine serum (FBS). THP-1 was maintained in RPMI-1640 medium with 10% FBS. All the cell culture media were supplemented with 1% penicillin/streptomycin (10378016, Gibco) to prevent bacterial contamination.THP-1 monocytes were conditioned with 5 nM PMA (P1585, Sigma) for 48 h to differentiate as THP1-derived macrophages. All cells were cultured at 37 °C in a humidified incubator with 5% CO_2_.

### Lentivirus transfection

The SLC1A5 knockdown or overexpression lentivirus was synthesized by Hanbio. For transfection, the cells were seeded and cultured with lentivirus for 16–18 h in six-well plates. Puromycin (0.5 μg/mL) was supplemented to select the transfected cells for a week. The efficacy of SLC1A5 knockdown or overexpression was tested by western blotting and Quantitative real-time PCR.

### Western Blotting (WB)

Frozen tissues and prepared cells were collected and lysed for quantification using a BCA protein assay kit (Thermo Fisher). Protein electrophoresis was followed by transferring to 0.22 μm PVDF membranes. After antibody incubation (SLC1A5, abcom, ab237704; SLC38A1, proteintech, 12039-1-AP; GPX4, abclonal, A1933; GAPDH, proteintech, 60004-1-Ig; HRP-conjugated goat anti-rabbit IgG, abclonal, AS063), final results were imaged by a near-infrared imaging system (Bio-Rad).

### Immunohistochemistry (IHC)

The paraffin sections were rehydrated with gradient ethanol solution. After antigen repair, the endogenous peroxidase activity was eliminated. The sections were blocked before incubation with the primary antibody (SLC1A5, abcom, ab237704; Anti -Iba1 Rabbit pAb, Servicebio, GB113502; Anti -CD86 Rabbit mAb, Servicebio, GB13585; Anti -CD163 Rabbit pAb, Servicebio, GB113152). SP Rabbit HRP Kit (DAB) (Cat.CW20355, CWBiotech) provides the protocol for the procedure. The results of IHC were scored according to the German Immunohistochemical Scores, and staining intensity at or above 4 points was defined as positive expression [47].

### Quantitative real-time PCR (qPCR)

Total cellular RNA was extracted by Trizol (Thermol Fisher). After reverse transcription of RNA into cDNA using Revert AidTM First Strand cDNA Synthesis Kit (Roche), qPCR was measured by using SYBR Green Master Mix (Roche). In order to ensure normalization, we selected β-actin as an internal control. The primer sequences were shown in Supplementary Table 3.

### Cell proliferation assay

Cell proliferation was detected by cell counting kit-8 (CCK8) and EDU assays. In the CCK8 assay, we seeded 2 × 10^3^ cells per well in a 96-well plate and cultured for 0–5 days. Cell viability was measured after adding 10 μL CCK-8 reagent for 2 h. We conducted an EdU staining proliferation assay, using BeyoClickTM EdU Cell Proliferation Kit (Beyotime). Cells were seeded to confocal dishes and cultured overnight. On the next day, cells were incubated for 2 h at a concentration of 10 μM EdU. The cells were then fixed, permeabilized and blocked following the manufacturer’s protocol. The nuclei were stained with DAPI.

### Colony formation assay

In the colony formation assay, 200 cells per well were grown in 6-well plates and cultured for 2 weeks. 4% polyoxymethylene was used to fix the cells. After washing, we stained the cells with 0.1% crystal violet, and photographs were then taken.

### Cell migration assay

5 × 10^4^ cells were transplanted into the upper chamber of the transwell plates (8 μm pore size, 6.5 mm diameter) with 100 μL of DMEM without FBS, while the bottom chamber was filled with 500 μL of 20% FBS DMEM. After 48 h incubation, upper surface cells were removed and lower surface cells were fixed with 4% paraformaldehyde and stained with Crystal Violet. After the removal of excess dye, we used an optical microscope to observe the cell and counted the cells throughout the filter.

### Hematoxylin-eosin (HE) staining

The mice brains were collected, fixed overnight with 4% paraformaldehyde, and dehydrated with 30% sucrose. The tissues were then sliced into 20μm sections for staining. Cell nuclei were stained with 1% Eosin (Solarbio) for 10 min. The cytoplasm was stained by hematoxylin (Solarbio) for 2 min. To selectively remove the excess dye, the sections were dipped in 1% acid alcohol for a few seconds and then sealed with neutral resin.

### Tumor xenografts transplantation

All animal experiments were approved by the Committee for Experimental Animal Use and Care of the Fourth Military Medical. This study followed the National Guidelines for the Experimentation of Animals in all animal experiments. After intraperitoneal injection of 10% chloral hydrate to anesthetize mice, the mice were implanted with 5 × 10^6^ tumor cells using a stereotactic head frame at a location 2 mm lateral and 0.5 mm anterior to bregma and 3 mm deep (right brain striatum). After 10 days later, mice were executed to isolate brain tissue and observe tumor growth. BALB/c nude mice (6–8w, male) were transplanted in situ with U87 cell line in which the expression of SLC1A5 was interfered (overexpressed or knocked down) by lentivirus and each group contained 5 mice. C57BL/6 mice (6–8w, male) were transplanted with the GL261 cell line. In the following treatment, the tumor-bearing mice received intraperitoneal injection of V9302 (30 mg/kg/day), Erastin (20 mg/kg/day), PD-1 mAb (10 mg/kg/2 days) or the same dose of saline and each group contained 5 mice. One batch of mice was executed 10 or 11 days after in situ tumor formation to remove the brain and observe the tumor size, and another batch was used to record their survival time. The molecular weights of all the drugs injected are less than 600 Daltons and can cross through the blood-brain barrier [48].

### Malondialdehyde (MDA) and glutathione (GSH) detection assay

The intracellular MDA levels were assessed by an MDA colorimetric kit (Jiancheng, Nanjing, China) following the manufacturer’s protocol. The intracellular GSH levels were assessed with GSH colorimetric assay kit (Solarbio, Beijing, China) following the manufacturer’s protocol.

### Reactive oxygen species (ROS) and peroxides (LPO) detection assay

DHFC-DA probe was used to measure reactive oxygen species (ROS) and C11 BODIPY 581/591 sensor (Invitrogen) was used to measure lipid peroxides (LPO) in the cells. In complete growth medium at 37 °C, cells were plated on confocal dishes and preincubated with 10 μmol DHFC-DA probe and C11 BODIPY 581/591 sensor for 30 min. Cells were stained with DAPI during the last 30 min of compound incubation. Following phosphate buffered saline (PBS) washing for 3 times, the cells were imaged by the confocal microscope (Nikon, Japan).

### RNA extraction and library sequencing

Sample RNA was isolated and purified using Trizol. mRNA was specifically captured, fragmented at high temperatures, and cDNA was synthesized by reverse transcriptase, which converted these complex duplexes of DNA and RNA into DNA duplexes with the ends of the duplex DNA braided into flat ends. An A base was added to each end of the duplex so that it could be joined to a joint with a T base at the end, and then the fragment size was screened and purified. The double strand was then digested and a library with a fragment size of 300 bp ± 50 bp was formed by PCR. At last, we performed the 2 × 150 bp paired-end sequencing on an Illumina Novaseq™ 6000 following the vendor’s recommended protocol.

### Tandem Mass Tag (TMT) labeled proteomics

We extracted total proteins from cell samples and performed the quality test on the proteins. Proteins were labeled with TMT and separated by liquid chromatography with peptide fraction separation, followed by Liquid chromatography-mass spectrometry (LC-MS) /mass spectrometry (MS) analysis and finally protein identification, quantification and functional analysis.

### Flow cytometry

After co-culturing with the glioma cells, the THP-1 derived macrophages were incubated at 4 °C for 30 min with fluorescein-conjugated specific antibodies against CD11b (BD Pharmingen, 550993) and CD206 (Invitrogen, 2324842). After washing twice with staining buffer, cells were resuspended in 300 μL of PBS with 1% FBS and analyzed using an ACEA NovoCyte. Results were processed and visualized with NovoExpress 1.5.6.

### Bioinformatic analysis

All bioinformatic analysis was conducted with R 4.0.3 (https://www.r-project.org/). Differentially expressed genes (DEGs) analysis and independent prognostic analysis were conducted to identify differentially expressed FRGs related to prognosis. The least absolute shrinkage and selection operator (LASSO) regression analysis was conducted to establish a prognostic model in TCGA cohort. Gene ontology (GO) and gene set enrichment analysis (GSEA) were used for function analysis. CIBERSORT and single sample GSEA (ssGSEA) were performed to evaluate the ratio of immune-infiltration cells.

### Statistical analysis

The exact value of the sample size (n) displayed in the figure legend represents the number of animals or cell culture. No statistical methods were used to predetermine sample sizes. However, our sample sizes were learned from previous studies [40, 49]. We randomly assigned animals to treatment groups according to a set of random numbers generated by Excel, and all analyses were completed by investigators who were blinded to the experimental groups. Data in this study are presented as the mean ± standard deviation from at least three independent experiments. SPSS 22.0 software and R 4.0.3 (https://www.r-project.org/) were used for statistical analysis. Differences between groups were examined by Student’s t-test or one-way ANOVA. *P* < 0.05 was considered statistically significant.

## Supplementary information

The agreement of the co-authors to the change of author list.

Checklist

Supplementary Figure Legend

Supplementary Figure 1

Supplementary Figure 2

Supplementary Figure 3

Supplementary Figure 4

Supplementary Figure 5

Supplementary Figure 6

Supplementary Figure 7

Supplementary Figure 8

Supplementary Figure 9

Supplementary Figure 10

Supplementary Western Blot

Supplementary Table 1

Supplementary Table 2

Supplementary Table 3

## Data Availability

The raw data supporting the conclusions of this article will be made available by the authors, without undue reservation. The TCGA database for this study can be downloaded from https://portal.gdc.cancer.gov/. The CGGA database for this study can be downloaded from http://www.cgga.org.cn/. The Rembrandt database for this study can be downloaded from https://www.britannica.com/biography/Rembrandt-van-Rijn. The immune-related data were downloaded from https://www.immport.org/home.
