# Supplementary material for: SLC1A5 enhances malignant phenotypes through modulating ferroptosis status and immune microenvironment in glioma
Source: Cell Death Dis. 2022 Dec 24;13(12):1071. doi: 10.1038/s41419-022-05526-w (PMC9789994; doi:10.1038/s41419-022-05526-w)
Supplement: Supplementary file 3 — Supplementary Figure Legend [file 41419_2022_5526_MOESM3_ESM.pdf]

Supplementary Figure Legend

Supplementary Figure 1 (A-F) The reliability of the risk score was verified in CGGA-325 cohort. (A) The relationship between the risk score and WHO grade. **(B)** The relationship between the risk score and IDH mutation status. **(C)** Patients were divided into high-risk and low-risk groups based on the median risk score. **(D)** The relationship between survival state and risk score. **(E)** Kaplan–Meier survival analysis for the survival time of high-risk patients and low-risk patients. **(F)** The ROC curves and AUC of the risk score in CGGA-325 cohort.

(G-L) The reliability of the risk score was verified in CGGA-693 cohort. (G) The relationship between the risk score and WHO grade. **(H)** The relationship between the risk score and IDH mutation status. **(I)** Patients were divided into high-risk and low-risk groups based on the median risk score. **(J)** The relationship between survival state and risk score. **(K)** Kaplan–Meier survival analysis for the survival time of high-risk patients and low-risk patients. **(L)** The ROC curves and AUC of the risk score in CGGA-693 cohort.

(M-Q) The reliability of the risk score was verified in Rembrandt cohort. (M) The relationship between the risk score and WHO grade. **(N)** Patients were divided into high-risk and low-risk groups based on the median risk score. **(O)** The relationship between survival state and risk score. **(P)** Kaplan–Meier survival analysis for the survival time of high-risk patients and low-risk patients. **(Q)** The ROC curves and AUC of the risk score in Rembrandt cohort.

Supplementary Figure 2 The ferroptosis-related risk score was associated with immuno-suppressive microenvironment in TCGA cohort. (A-B) GO enrichment analysis of the relationship between the risk score and the immune-related biological processes (**A**, bar plot; **B**, pot plot). **(C)** CIBERSORT analysis of the infiltration of immune cells between high-risk and low-risk groups **(D-E)** ssGSEA analysis of the relationship between the risk score and the immune-cell scores**(D),** and immunity-related pathways**(E)**. **(F-H)** The association of immune scores, tumor purity and risk scores in TCGA cohort.

Supplementary Figure 3 The relationship between the expression of the genes in the model and WHO grade in TCGA, CGGA-325, CGGA-693, Rembrandt cohorts.

(A) The relationship between CAPG and WHO grade in TCGA, CGGA-325, CGGA-693, Rembrandt cohorts.

(B) The relationship between FANCD2 and WHO grade in TCGA, CGGA-325, CGGA-693, Rembrandt cohorts.

(C) The relationship between HMOX1 and WHO grade in TCGA, CGGA-325, CGGA-693, Rembrandt cohorts.

(D) The relationship between HSPB1 and WHO grade in TCGA, CGGA-325, CGGA-693, Rembrandt cohorts.

(E) The relationship between RRM2 and WHO grade in TCGA, CGGA-325, CGGA-693, Rembrandt cohorts.

(F) The relationship between STEAP3 and WHO grade in TCGA, CGGA-325, CGGA-693, Rembrandt cohorts.

Supplementary Figure 4 The relationship between the expression of the genes in the model and survival time in TCGA, CGGA-325, CGGA-693, Rembrandt cohorts.

(A) The relationship between CAPG expression and survival time in TCGA, CGGA-325, CGGA-693, Rembrandt cohorts.

(B) The relationship between FANCD2 expression and survival time in TCGA, CGGA-325, CGGA-693, Rembrandt cohorts.

(C) The relationship between HMOX1 expression and survival time in TCGA, CGGA-325, CGGA-693, Rembrandt cohorts.

(D) The relationship between HSPB1 expression and survival time in TCGA, CGGA-325, CGGA-693, Rembrandt cohorts.

(E) The relationship between RRM2 expression and survival time in TCGA, CGGA-325, CGGA-693, Rembrandt cohorts.

(F) The relationship between STEAP3 expression and survival time in TCGA, CGGA-325, CGGA-693, Rembrandt cohorts.

Supplementary Figure 5 The expression of SLC1A5 correlated with WHO grade and survival time of glioma patients in CGGA-325, CGGA-693, Rembrandt cohorts. (A-B) The relationship between SLC1A5 expression and WHO grade **(A)** and survival time **(B)** in CGGA-325 cohort. **(C-D)** The relationship between SLC1A5 expression and WHO grade **(C)** and survival time **(D)** in CGGA-693 cohort. **(E-F)** The relationship between SLC1A5 expression and WHO grade **(E)**, and survival time **(F)** in Rembrandt cohort.

Supplementary Figure 6 The expression of SLC1A5 in normal astrocyte and glioma cell lines. (A) The protein expression of SLC1A5 and SLC38A1 in the normal human astrocyte cell line, HA1800, and the glioma cell lines. **(B)** The relative mRNA expression in the normal human astrocyte cell line, HA1800, and the glioma cell lines.

Supplementary Figure 7 The validation of the lentivirus transfection in T98G cells. (A) The WB results showing the protein expression of SLC1A5 in T98G cells after lentiviral transduction. **(B)** The qPCR results showing the relative mRNA expression of SLC1A5 in T98G cells after lentiviral transduction.

Supplementary Figure 8 SLC1A5 overexpression promoted the proliferation and invasion of glioma cells *in vitro*. (A) The CCK-8 assay results of cell viability after SLC1A5 overexpression. **(B)** The EdU assay results of proliferative phase cells after SLC1A5 overexpression (Scale Bar =20 μm). **(C)** The colony formation results of T98G cells after SLC1A5 overexpression. **(D)** The transwell assay results of invasive cells after SLC1A5 overexpression (Scale Bar=20 μm).

Supplementary Figure 9 SLC1A5 overexpression reduced oxidative stress and ferroptosis status in glioma cells. (A) The CCK-8 assay results of cell viability after SLC1A5 overexpression with Erastin treatment. **(B)** The MDA level after SLC1A5 overexpression with Erastin treatment. **(C)** The level of reduced GSH after SLC1A5 overexpression with Erastin treatment. **(D)** The fluorescence intensity of DHFC-DA (green) in SLC1A5 overexpression T98G cells with Erastin treatment (Scale Bar = 20 μm). **(E)** The level of oxidized lipid peroxides(green) in SLC1A5 overexpression T98G cells with Erastin treatment (Scale Bar = 20 μm).

Supplementary Figure 10 Pharmacological inhibition of SLC1A5 enhanced intracellular level of oxidative stress and ferroptosis status. (A) The MDA level of T98G cell line in V9302 and control groups. **(B)** The reduced GSH level of T98G cell line in V9302 and control groups. **(C, E)** The fluorescence intensity of DHFC-DA (green) after V9302 treatment in T98G cells (Scale Bar=20 μm). **(D, F)** The level of oxidized lipid peroxides(green) after V9302 treatment in T98G cells (Scale Bar=20 μm).
