# Supplementary figures and images for: SLC1A5 enhances malignant phenotypes through modulating ferroptosis status and immune microenvironment in glioma
Source: Cell Death Dis. 2022 Dec 24;13(12):1071. doi: 10.1038/s41419-022-05526-w (PMC9789994; doi:10.1038/s41419-022-05526-w)

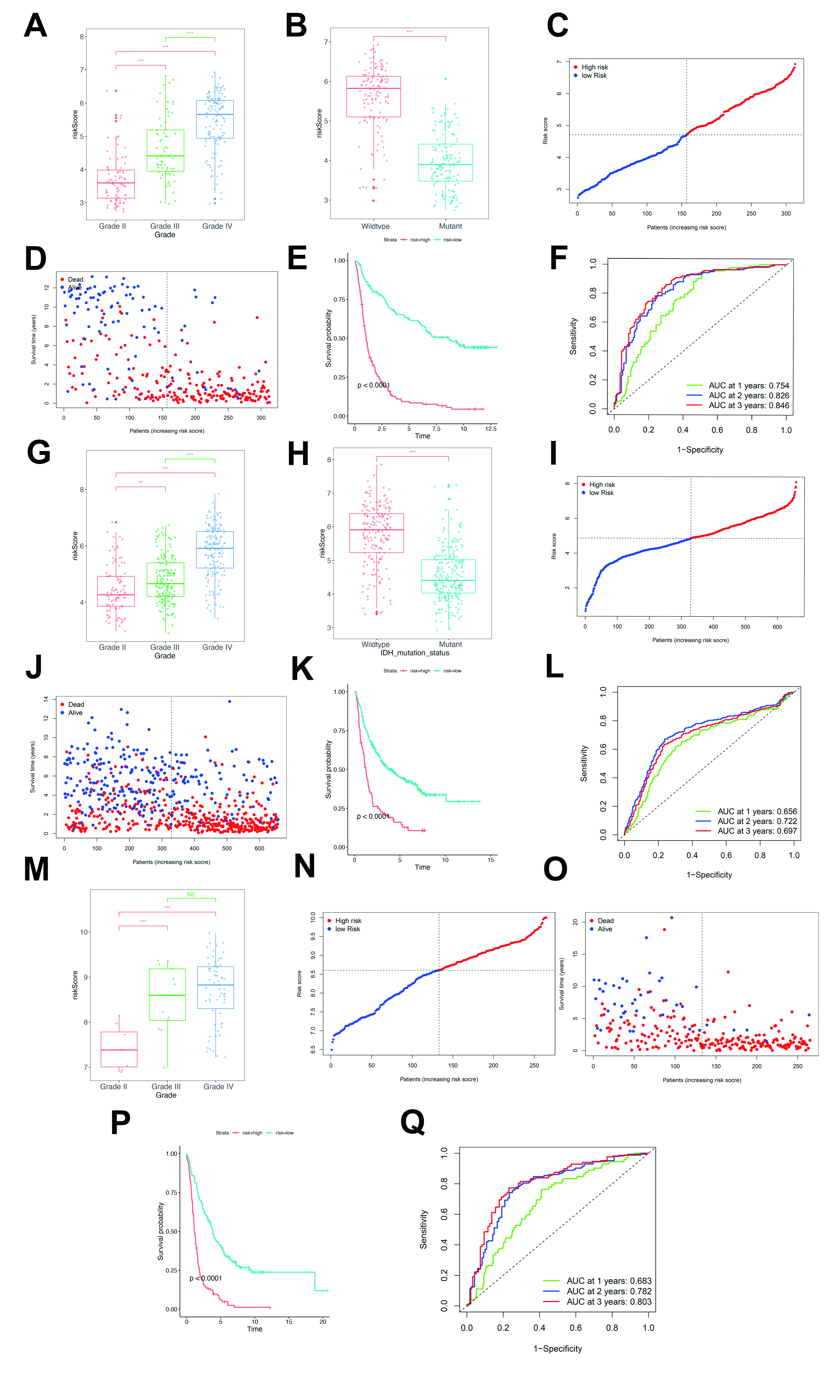

Supplement: Supplementary file 4 — Supplementary Figure 1 [file 41419_2022_5526_MOESM4_ESM.jpg]

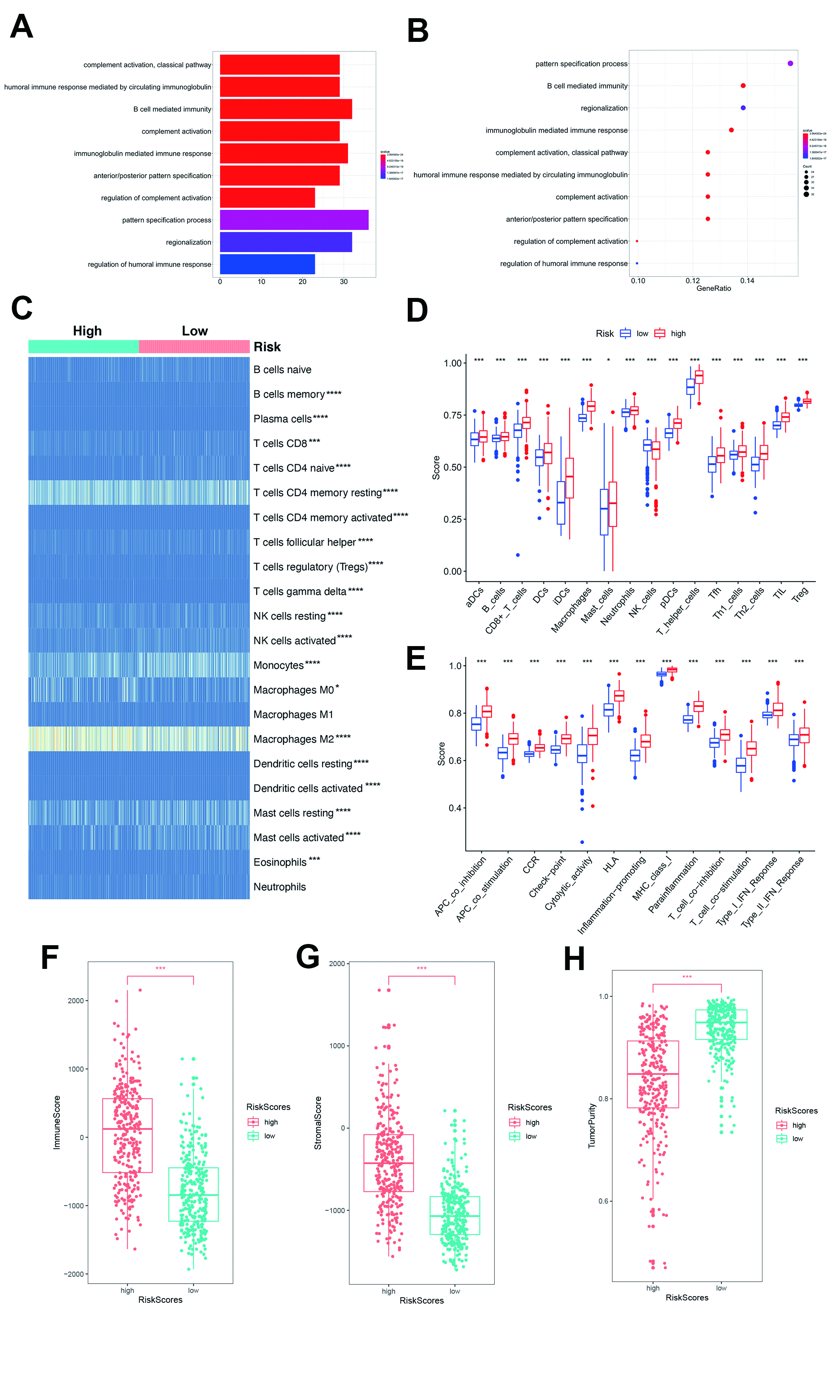

Supplement: Supplementary file 5 — Supplementary Figure 2 [file 41419_2022_5526_MOESM5_ESM.jpg]

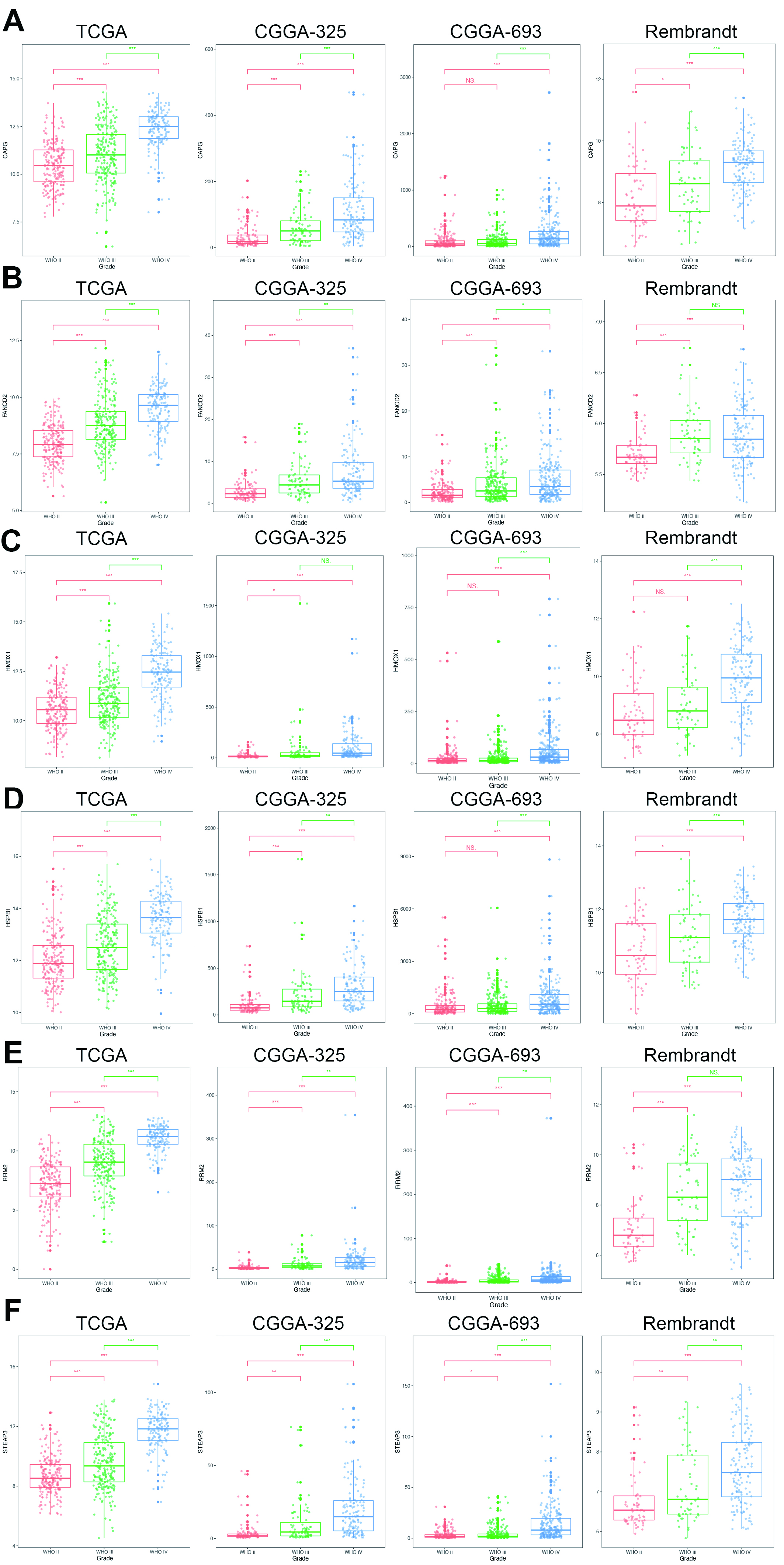

Supplement: Supplementary file 6 — Supplementary Figure 3 [file 41419_2022_5526_MOESM6_ESM.jpg]

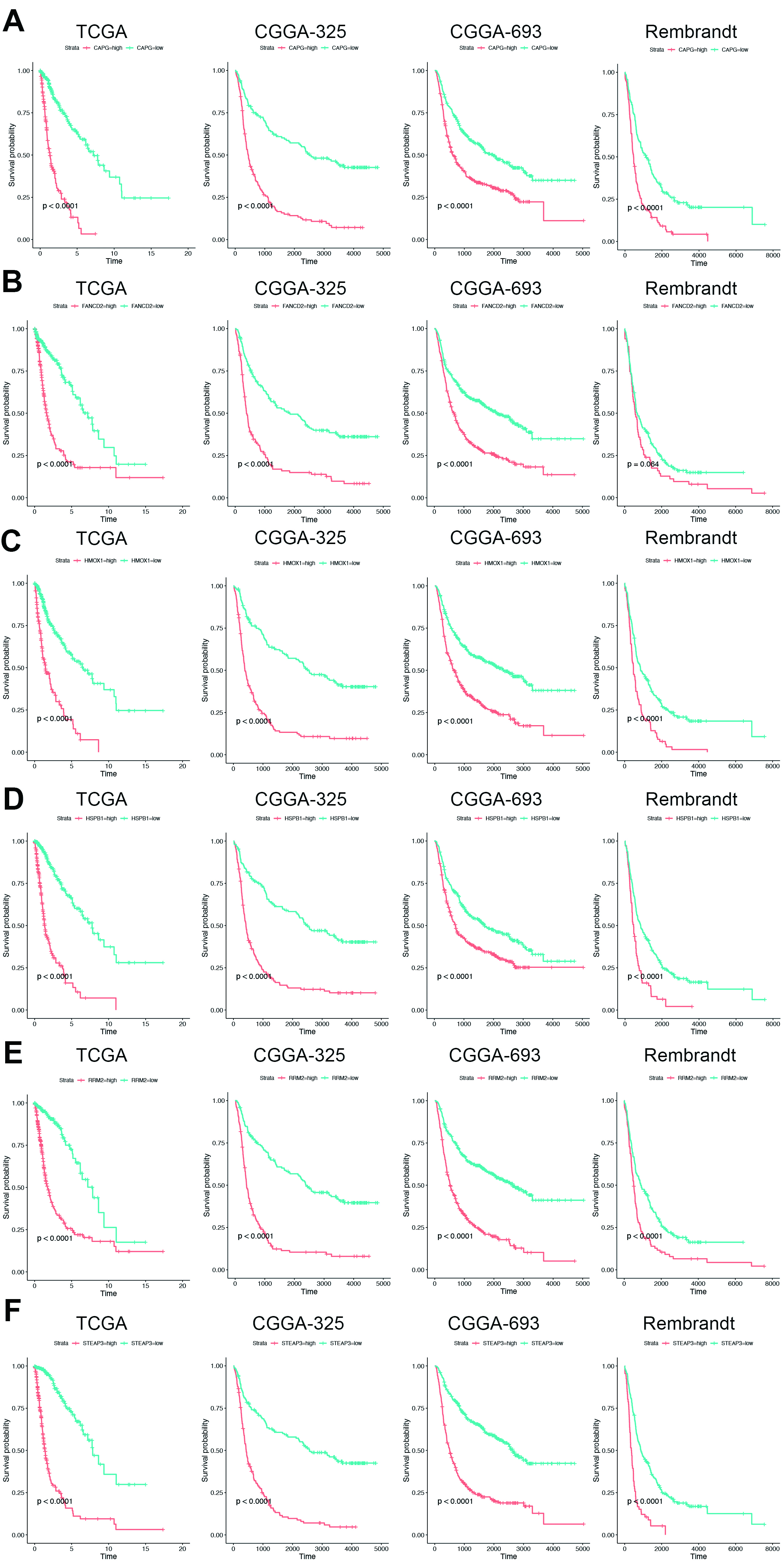

Supplement: Supplementary file 7 — Supplementary Figure 4 [file 41419_2022_5526_MOESM7_ESM.jpg]

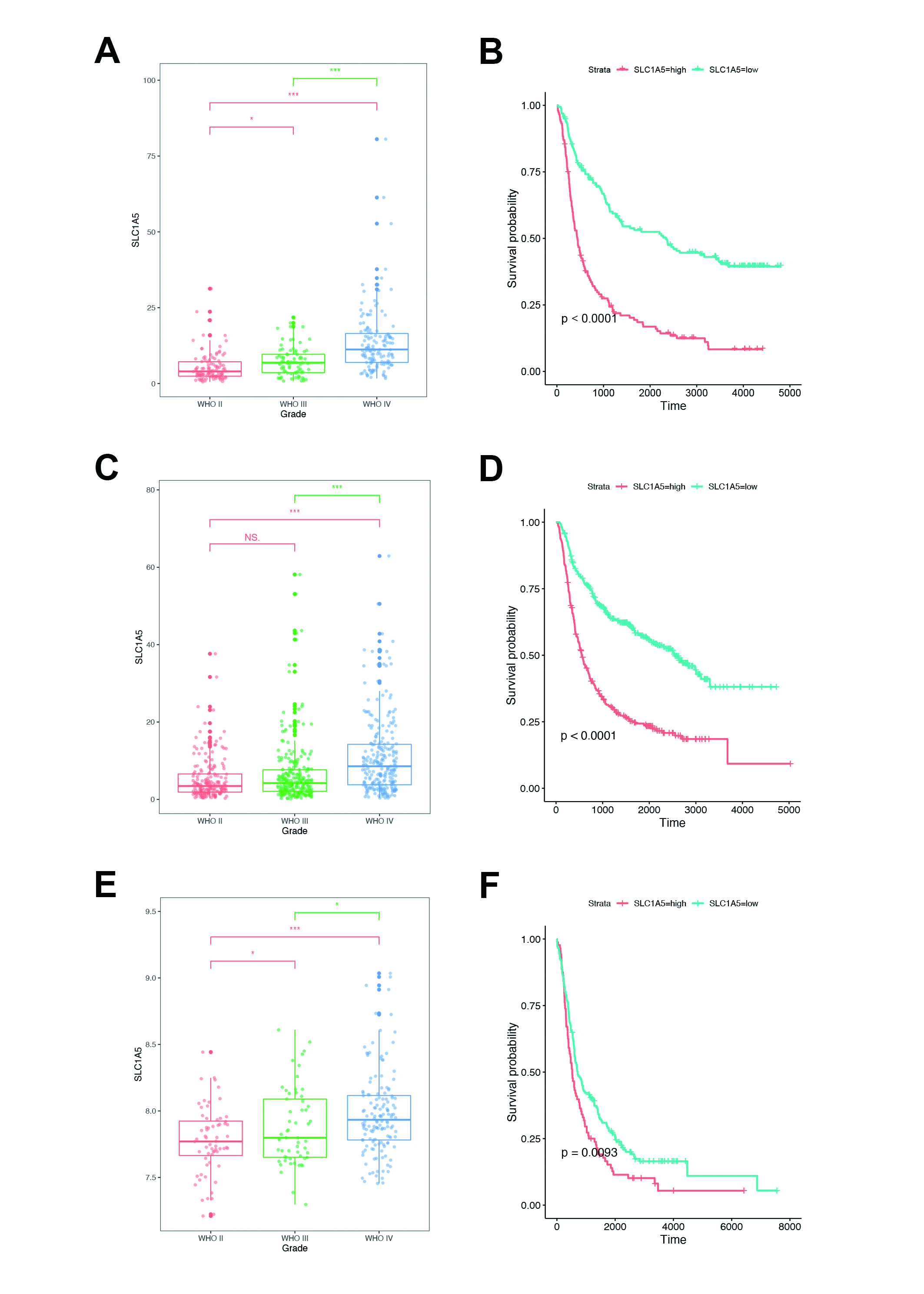

Supplement: Supplementary file 8 — Supplementary Figure 5 [file 41419_2022_5526_MOESM8_ESM.jpg]

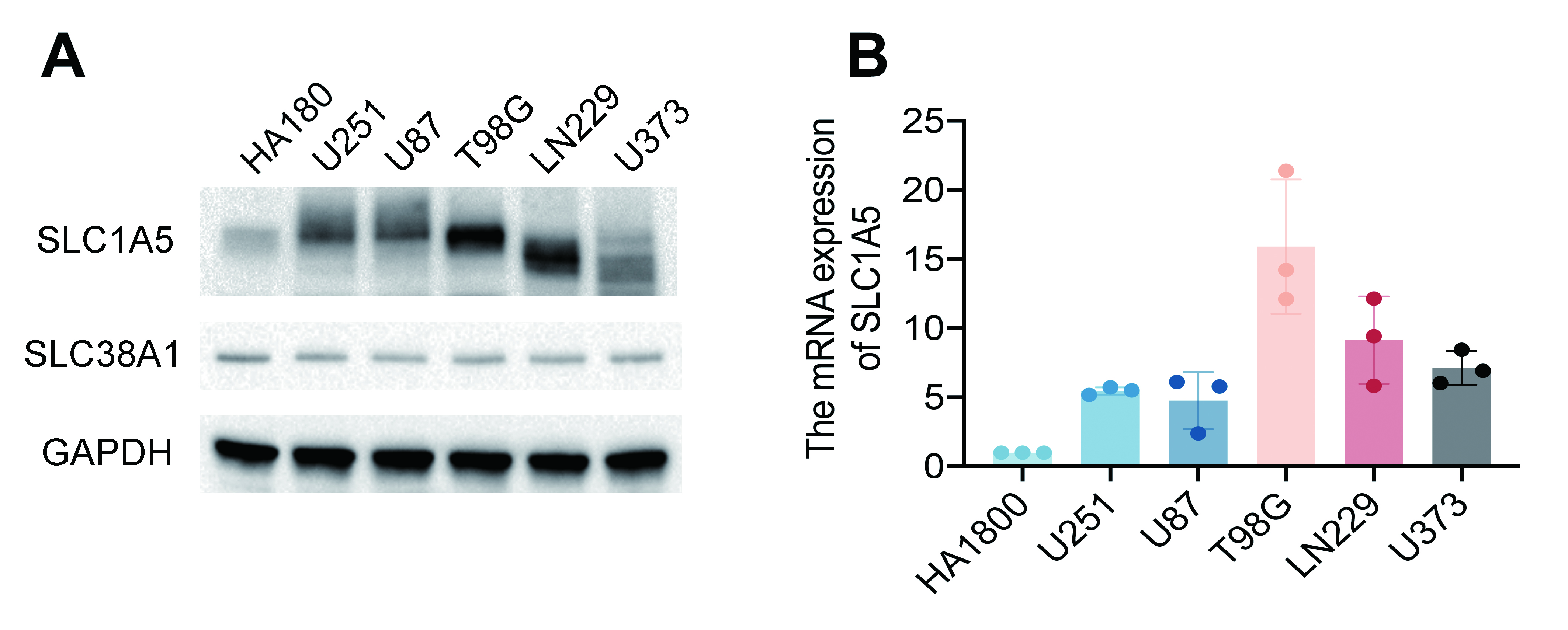

Supplement: Supplementary file 9 — Supplementary Figure 6 [file 41419_2022_5526_MOESM9_ESM.jpg]

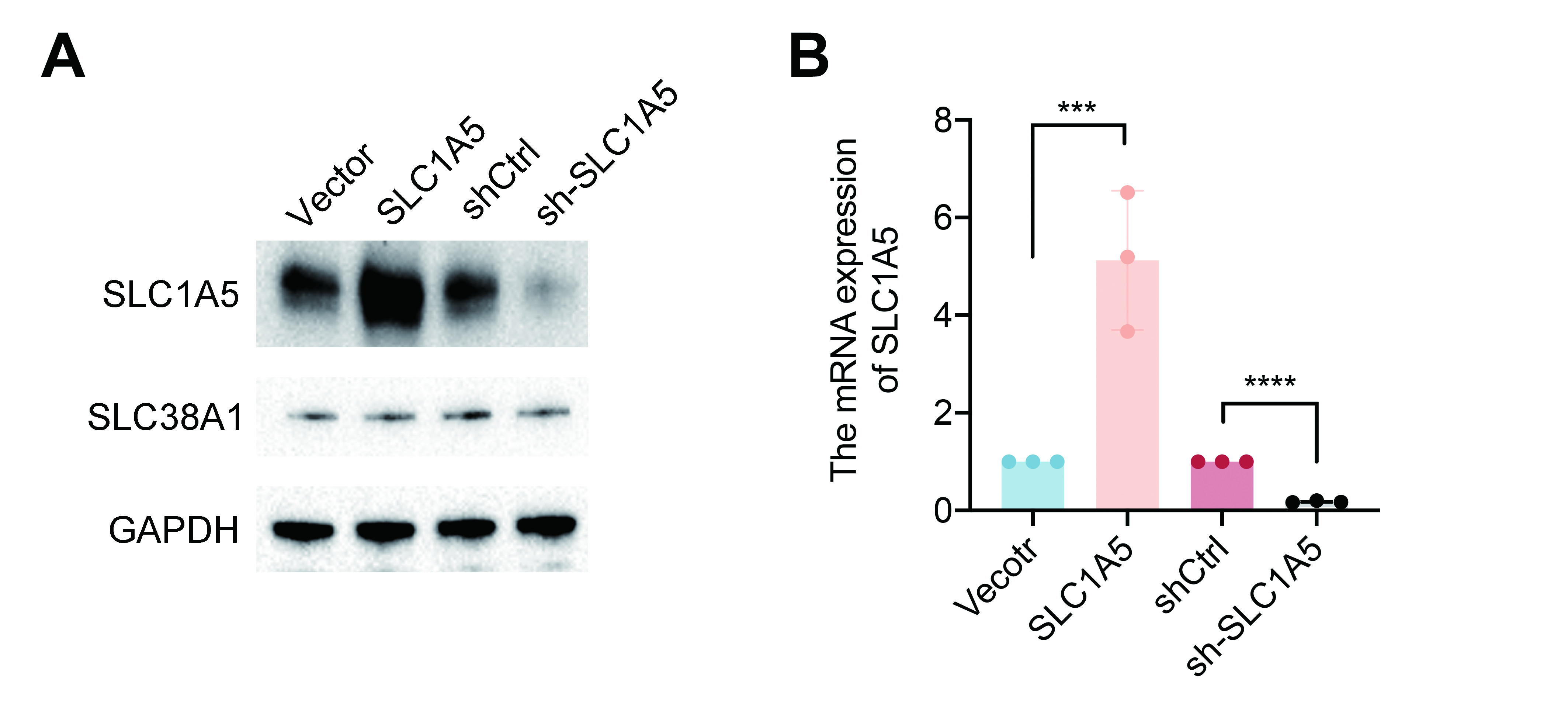

Supplement: Supplementary file 10 — Supplementary Figure 7 [file 41419_2022_5526_MOESM10_ESM.jpg]

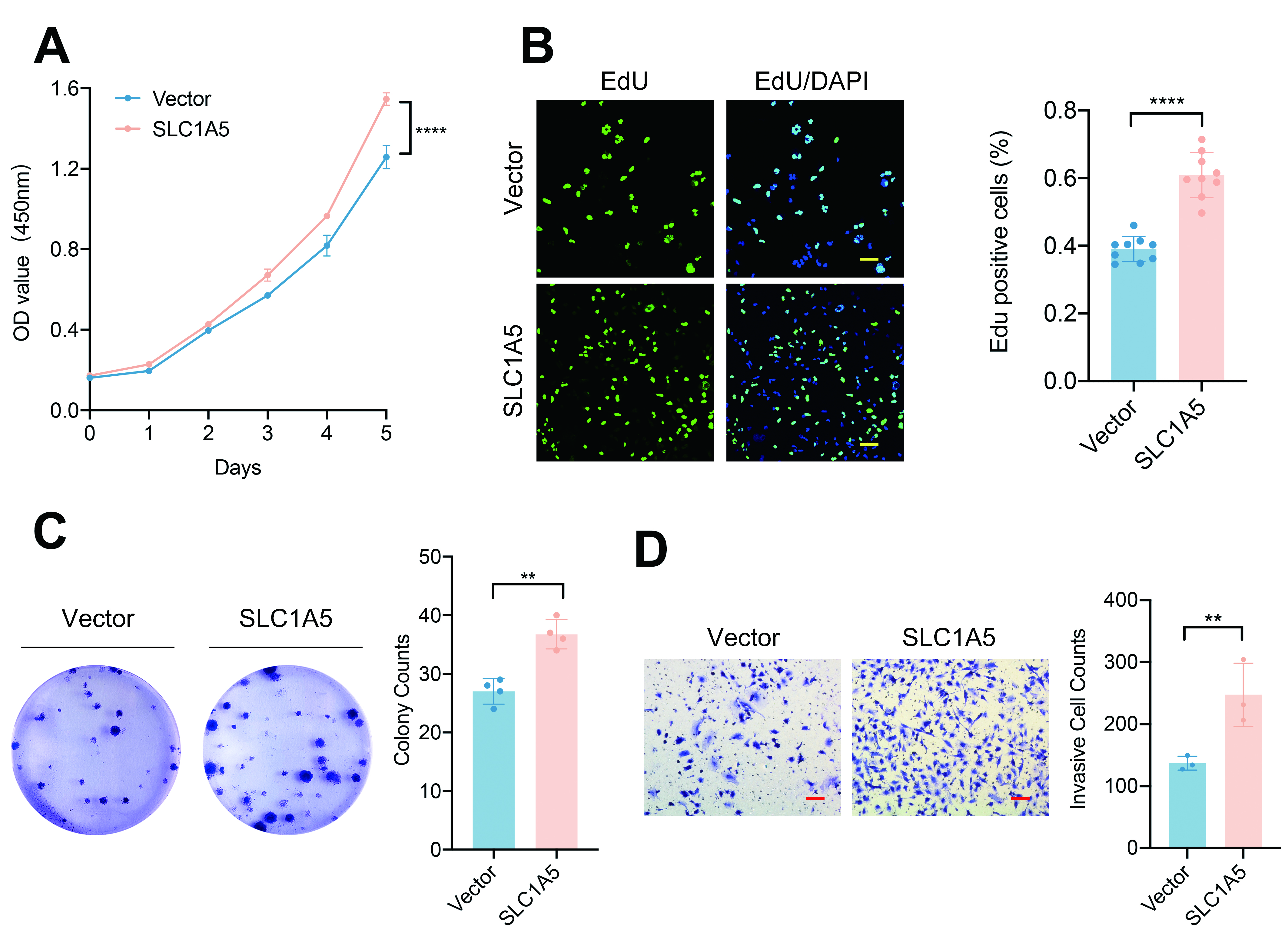

Supplement: Supplementary file 11 — Supplementary Figure 8 [file 41419_2022_5526_MOESM11_ESM.jpg]

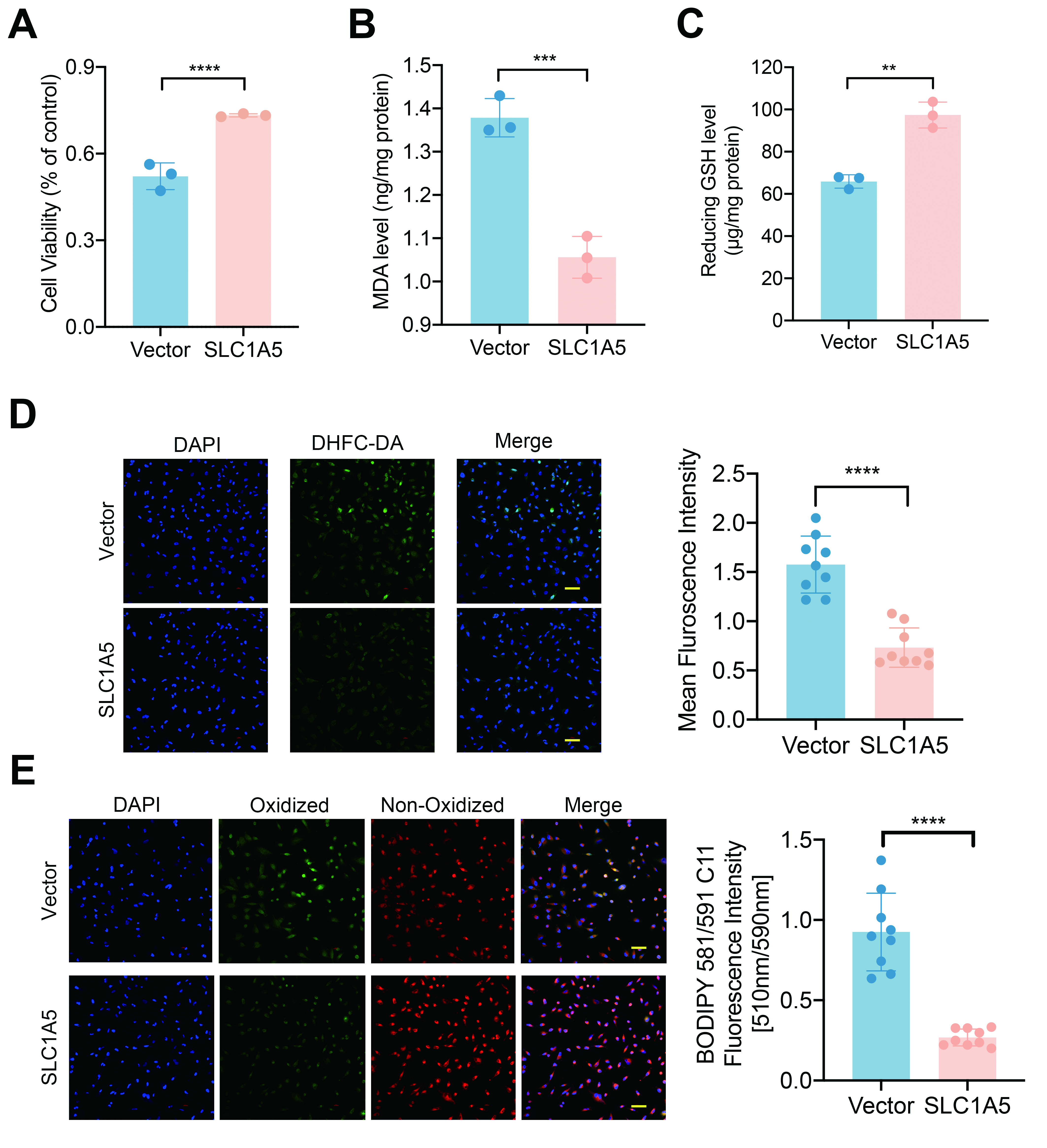

Supplement: Supplementary file 12 — Supplementary Figure 9 [file 41419_2022_5526_MOESM12_ESM.jpg]

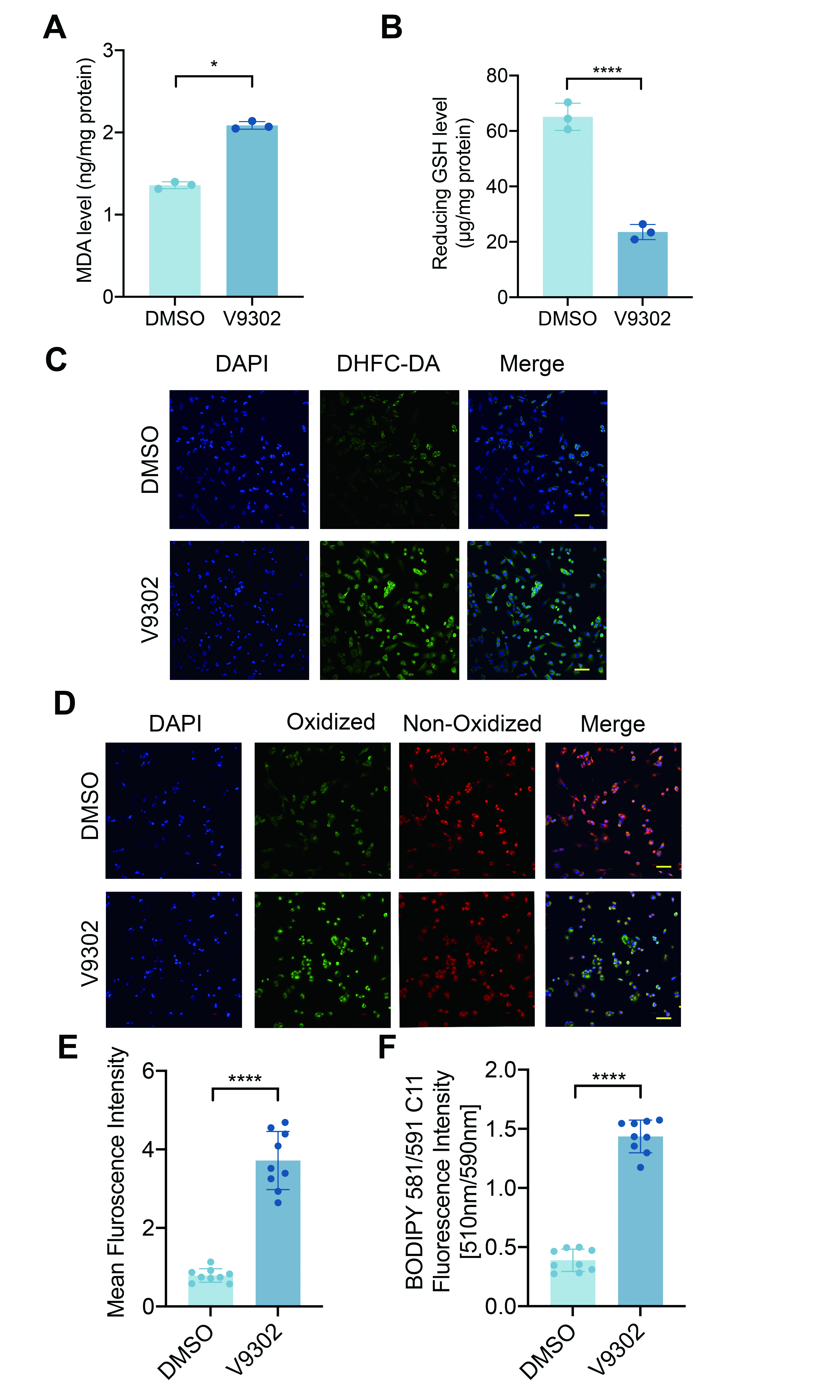

Supplement: Supplementary file 13 — Supplementary Figure 10 [file 41419_2022_5526_MOESM13_ESM.jpg]

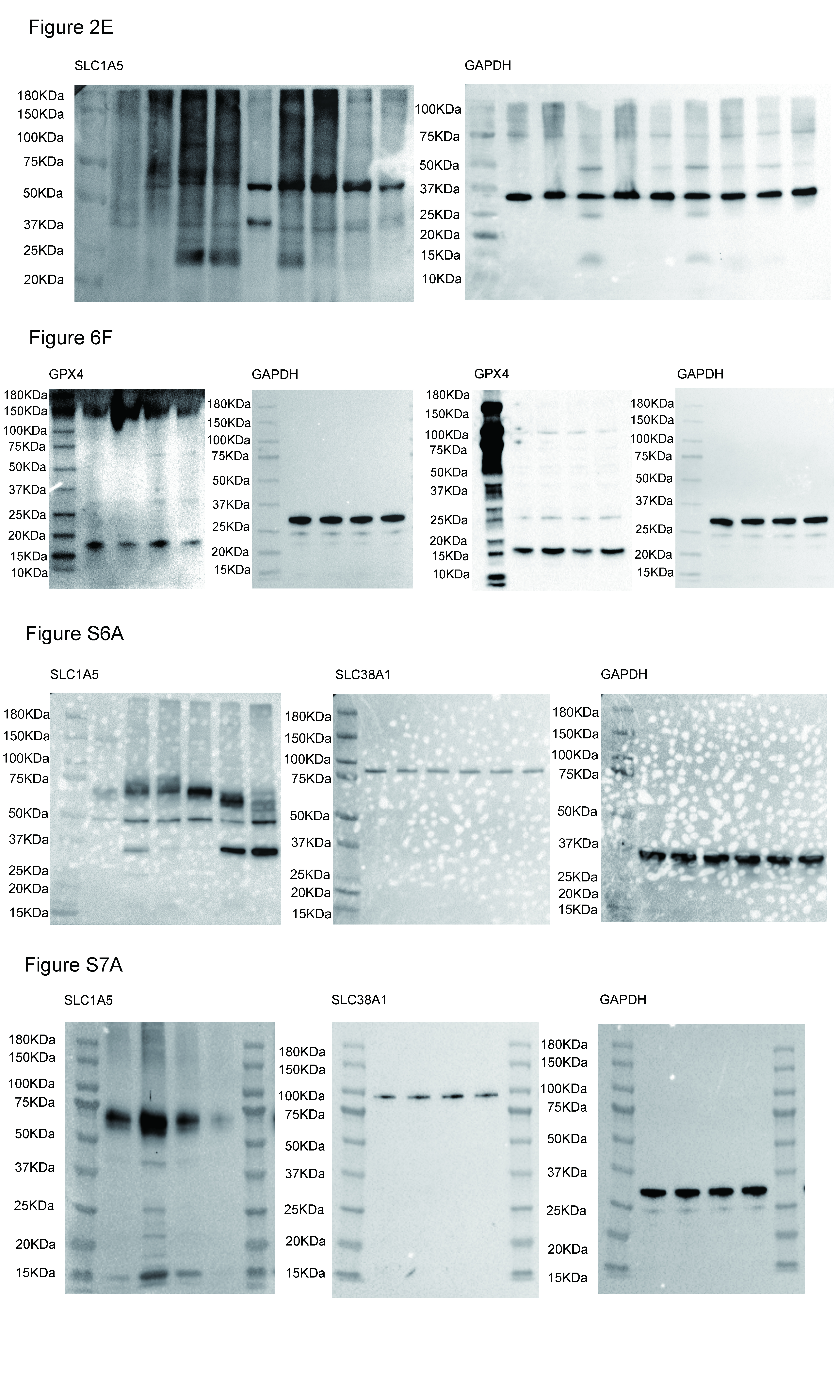

Supplement: Supplementary file 14 — Supplementary Western Blot [file 41419_2022_5526_MOESM14_ESM.jpg]
